# Multiple potential recombination events among Newcastle disease virus genomes in China between 1946 and 2020

**DOI:** 10.3389/fvets.2023.1136855

**Published:** 2023-05-03

**Authors:** Amina Nawal Bahoussi, Pir Tariq Shah, Jia-Qi Zhao, Pei-Hua Wang, Yan-Yan Guo, Changxin Wu, Li Xing

**Affiliations:** ^1^Institutes of Biomedical Sciences, Shanxi University, Taiyuan, China; ^2^Department of Bioengineering, College of Life Science, Shanxi University, Taiyuan, China; ^3^Shanxi Provincial Key Laboratory of Medical Molecular Cell Biology, Shanxi University, Taiyuan, China; ^4^Shanxi Provincial Key Laboratory for Prevention and Treatment of Major Infectious Diseases, Taiyuan, China; ^5^The Key Laboratory of Chemical Biology and Molecular Engineering of Ministry of Education, Shanxi University, Taiyuan, China

**Keywords:** Newcastle disease virus, recombination, phylogeography, oncolytic, attenuated vaccine

## Abstract

**Introduction:**

Newcastle Disease Virus (NDV) is a highly adaptable virus with large genetic diversity that has been widely studied for its oncolytic activities and potential as a vector vaccine. This study investigated the molecular characteristics of 517 complete NDV strains collected from 26 provinces across China between 1946-2020.

**Methods:**

Herein, phylogenetic, phylogeographic network, recombination, and amino acid variability analyses were performed to reveal the evolutionary characteristics of NDV in China.

**Results and discussions:**

Phylogenetic analysis revealed the existence of two major groups: GI, which comprises a single genotype Ib, and GII group encompassing eight genotypes (I, II, III, VI. VII. VIII, IX and XII). The Ib genotype is found to dominate China (34%), particularly South and East China, followed by VII (24%) and VI (22%). NDV strains from the two identified groups exhibited great dissimilarities at the nucleotide level of phosphoprotein (P), matrix protein (M), fusion protein (F), and haemagglutinin-neuraminidase (HN) genes. Consistently, the phylogeographic network analysis revealed two main Network Clusters linked to a possible ancestral node from Hunan (strain MH289846.1). Importantly, we identified 34 potential recombination events that involved mostly strains from VII and Ib genotypes. A recombinant of genotype XII isolated in 2019 seems to emerge newly in Southern China. Further, the vaccine strains are found to be highly involved in potential recombination. Therefore, since the influence of recombination on NDV virulence cannot be predicted, this report’s findings need to be considered for the security of NDV oncolytic application and the safety of NDV live attenuated vaccines.

## 1. Introduction

Oncolytic virotherapy is a novel approach to cancer treatment that has demonstrated promising benefits (1). Oncolytic viruses replicate selectively in the tumor cells and engender an anti-tumoral effect through direct oncolysis or indirect enhancement of bystander effects via cytokine production, leading to tumor cell death (1). In the last decades, several viruses have been evaluated for clinical trials as oncolytic virotherapy, among which is the aetiological agent of new castle disease.

Newcastle disease (ND) is a highly infectious avian viral disease affecting a wide range of birds (2). ND was first identified in 1926 in Indonesia and in 1927 in New Castle upon Tyne, England (3). Since then, extensive ND outbreaks have been reported in many countries, including Korea, the Philippines, India, Sri Lanka, and Japan. ND is caused by Newcastle disease virus (NDV), a member of the *Avian orthoavulavirus 1* (AOAV-1) species from the *Orthoavulavirus* genus of the *Avulavirinae* subfamily within the *Paramyxoviridae* family [International Committee on Taxonomy of Viruses (ICTV), 2019^1^] (4).

NDV is an enveloped, non-segmented, negative-sense, single-stranded RNA genome of approximately ~15,000 nucleotides (nt) in length (5). NDV genome possesses six main genes encoding nucleoprotein (NP), phosphoprotein (P), matrix protein (M), fusion protein (F), haemagglutinin-neuraminidase (HN), and large polymerase protein (L) (6), in addition to two non-structural proteins (V and W) in virus-infected cells generated by RNA editing of P gene (7). The P gene encodes three proteins (P, V, and M), while the remaining five genes encode a single major protein (8–10). HN and F are surface glycoproteins mainly implicated in NDV pathogenesis. HN is involved in receptor binding and is in charge of activating the F protein to induce membrane fusion and facilitate virus entry (11, 12). HN is responsible of the virus attachment to the sialic-acid-containing receptors to prevent viral self-aggregation (13). The expression of the HN protein alters the conformation of the NDV F protein that is responsible for virus budding (14, 15). HN protein has been reported to determine the tropism (16). NDV virulence depends on amino acid differences in the HN protein (16). Therefore, HN-F proteins are implicated in determining the virulence of NDV (16, 17).

M protein is responsible for the virion assembly at the host cell membrane (18, 19). The N protein guards against nucleases in the genome and forms the nucleocapsid structure (20). P protein is essential for viral transcription and replication (viral RNA synthesis)and helps maintain the L protein’s stability in the P-L complex (20). In virulence, the P protein depends on the type of cell and NDV strain (20); the L protein influences NDV virulence and controls the rate of viral RNA synthesis during replication (21). Based on the clinical signs and pathogenicity in chicken, NDV is categorized into four pathotypes ranging from severe with a 100% mortality rate to asymptomatic forms: velogenic, mesogenic, lentogenic, and asymptomatic enteric (22), causing significant economic losses worldwide (23). A correlation between the pathogenic and oncolytic properties of NDV has been reported, where velogenic and mesogenic strains replicate more efficiently in human tumor cells.

Historically, NDV was phylogenetically classified based on the complete F gene sequences into two main classes: class I and class II (24). The NDV class I was further divided into nine genotypes, and class II into eighteen (I–XVIII) genotypes. Recently, in 2019, Dimitrov et al. proposed a unified updated NDV classification system in which Class I encompasses a single genotype subdivided into three further subgenotypes (1.1.1, 1.1.2, and 1.2), contrary to Class II, which is more diverse with twenty genotypes (I to XXI) and containing most of the ND outbreak reported strains (25, 26).

In China, the first ND outbreak occurred probably at the same time as the initial ND global epidemic, dating back to the 1920s; however, the cause was not identified until 1946 (27). Since the third ND pandemic in late 1970 to 1980, eight genotypes of Class II, including I, II, III, VI, VII, VIII, IX and XII, have been isolated in China. According to the epidemiological data, the VII genotype has been strongly linked to large outbreaks in China, particularly between 2000 and 2015 (28).

Since the first observation of the NDV oncolytic effects in 1965 (29), the virus has been tested in various animal and human models for anti-tumor activity (30–33). Currently, NDV is well known to be used as a potent Oncolytic virotherapy (OV). NDV has been reported as a genetically stable, easy to manipulate, modular genome, and low recombination rate, making it a promising vaccine vector for veterinary and human pathogens (34).

Up to now, NDV is one of the four species of OV anti-tumor agents that were most used in China, in addition to Herpes Simplex virus (HSV), adenovirus, and Alphavirus M1 stain (35, 36). However, despite decades of *in vitro* and *in vivo* research studies on the NDV oncolytic virotherapy advantages against cancer, few have been accepted for clinical trials in China compared to other countries in Europe and the US (37–39). Therefore, to close the translational gap and help Lab knowledge reach the bed-side, we reviewed, using bioinformatics, the genetic characteristic and genomic stability of China NDV complete sequences reported between 1946 and 2020, and uncover patterns of potential recombination in China-NDV strains evolution and transmission during the last decades across 26 provinces.

## 2. Materials and methods

### 2.1. Dataset compilation

To track the evolution of China NDV strains and their genetic diversity, we generated an updated complete genome sequence of all China NDV strains dataset available on the NCBI GenBank database where all available full-length genome sequences of ~15,000 nt in length were collected. After alignment, all identical sequences and missing sequences have been discarded. We evaluated a total of 517 China NDV full-length sequences identified from 26 different regions between 1946 and 2020. 133 out of 517 viruses were identified during 2001–2010 and 368 during 2011–2020. The NDV strains were recognized by their GenBank accession number, virus name, year, and country/region.

### 2.2. Construction of phylogenetic tree

All China-NDV complete sequences (517) were aligned with ClustalW using the Molecular Evolutionary Genetics Analysis 11 software (MEGA11) (40) and edited using BioEdit v 7.2.5. A Maximum Likelihood (ML) phylogenetic tree was constructed using the IQ-TREE multicore version 1.6.12 with the best-fitting model GTR + F + I + G4 and 1,000 bootstraps (41). The final dataset of the ML method consisted of 15,235 positions. The tree was visualized and modified using FigTree v1.4.

### 2.3. Similarity analysis

Genomic similarities between China NDV genotypes were determined using fourteen China NDV representative full-length genome sequences for each genotype of the two identified groups: GI and GII. The virus NDV/ Guangdong/05/2011 (GenBank ID: MT4478740) was involved as a Query strain. The genomic similarity plot was carried out using SimPlot ver.3.5.1 1 (42).

### 2.4. Phylogeographic network analysis

The phylogeographic network of the full-length genomic sequences of NDVs was inferred using the Minimum Spanning Network (MSN) implemented by PopArt v1.7 (43). The phylogeographic network included strains reported from 26 different regions of China (Table 1).

**Table 1:** Geographic distribution of NDV genotypes in China during 1946–2020.

NDV genotype	VII	XII	VI	VIII	III	IX	I	II	Ib
Hunan	18	2	10		1	2	1	4	5
Hubei	8		9			1			1
Jiangxi	3		6		1			1	4
Shandong	11		14	3	2	5			4
Jiangsu	6		1						
Taiwan	12		3			1		2	
Shanghai	4	8	6			2	2		4
Fujian	2		1						2
Anhui	22		13		1		3	5	22
Zhejiang	1		1						1
Beijing	1								
Hebei	4						1	2	1
Tianjin	7		21	1		1	3	3	5
Heilongjiang	6		1			1	4	1	
Jilin	2		6	1		5		1	
Liaoning	3		2						1
Shaanxi	2		2						
Gansu	1				1			5	
Qinghai	5		4				2		7
Ningxia	5		4						
Xinjiang			2		1		5	1	1
Yunnan			2						
Guizhou			2						2
Sichuan			2						1
Guangxi							10	1	61
Guangdong							5	5	52
Total	123	10	112	5	7	18	36	31	174

Central China (*N* = 82), East China (*N* = 196), North China(*N* = 41), Northeast China (*N* = 81), Northwest China (*N* = 32), Southwest China (*N* = 17), South China (*N* = 69).

### 2.5. Recombination analysis

After alignment, the NDV full-length genome sequences (~ 15,000 nt) were imported into the RDP4 software package. The occurrence of recombination was explored using the seven algorithms implemented in the RDP4 package, including RDP, GENECONV, Bootscan, MaxChi, Chimera, SiScan and 3seq. A recombination event was considered positive when supported by at least four of the aforementioned algorithms. The parental sequences and mapping of possible breakpoints were screened using SimPlot version 3.5.1. The authenticity of recombination was further verified using phylogenetic analysis.

### 2.6. Amino acids variability analysis

We retrieved separately the complete sequences of NDV ORFs encoding the F, HN, M, NP, P, and L proteins and were aligned separately with ClustalW using the MEGA11 software (40). The sequences were manually translated and edited using BioEdit v 7.2.5. The amino acids variability map was determined using the Wu-Kabat variability coefficient, implemented by the Protein Variability Server (PVS) (44), using the formula V = N*k/n, where V is the variability, *n* corresponds to the time that the most commonly recognized amino acid at that position is available, *k* represents the number of different amino acids at a given position and *N* is the number of sequences in the alignment (45).

## 3. Results

### 3.1. Newcastle disease virus Genotype Ib dominates East and Central China

**Figure 1:**
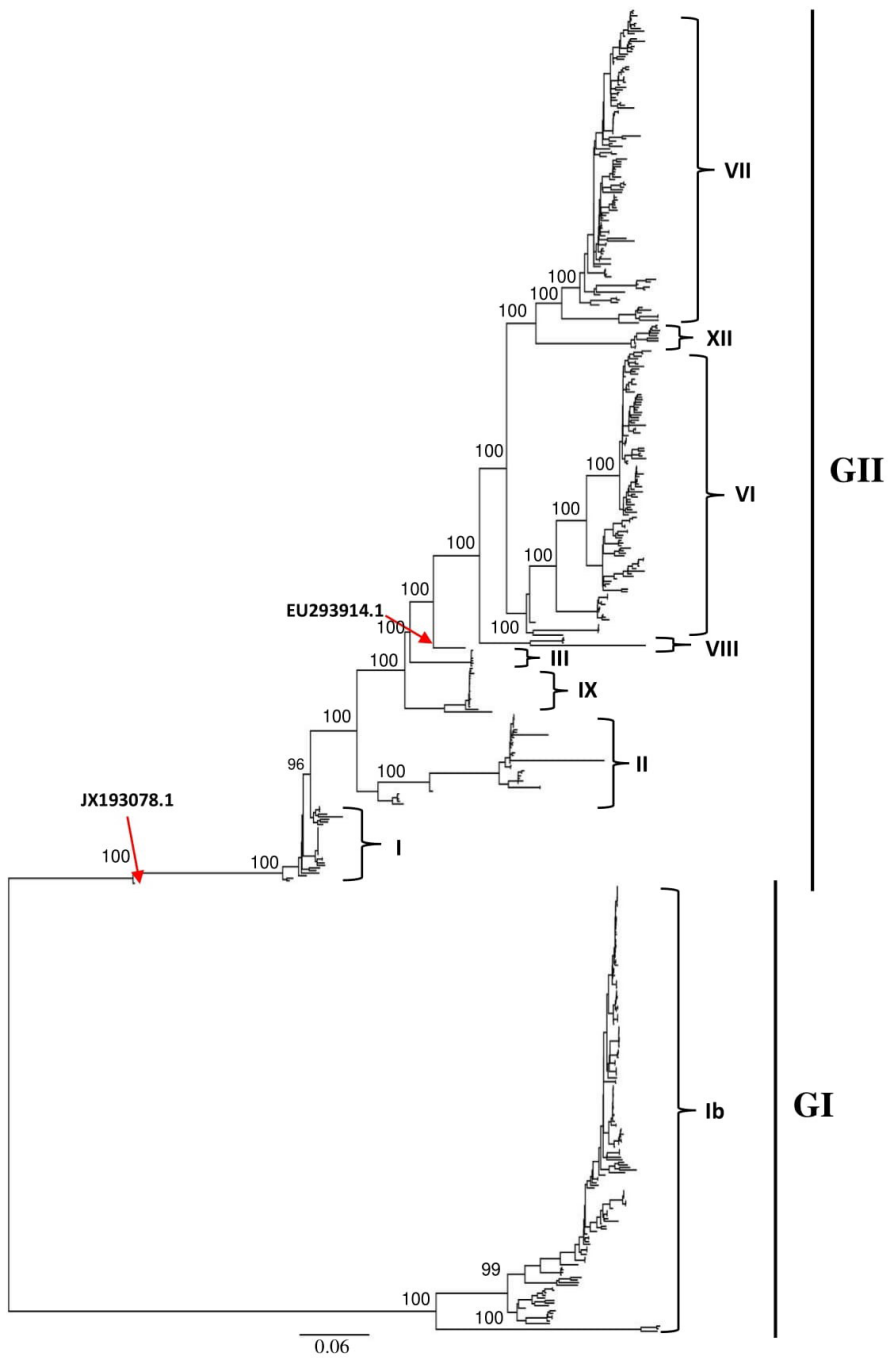
Phylogenetic analysis of the Newcastle disease viruses circulating in China. Phylogenetic relationship among 517 NDV strains was based on the complete genomic nucleotide sequences collected between 1946 and 2020. The NDV sequences were obtained from the NCBI GenBank database and the tree construction was done using the ML method with the best-fitting model GTR + F + I + G4 in the IQ-TREE multicore version 1.6.12 after 1,000 bootstrap replication.

**Figure 2:**
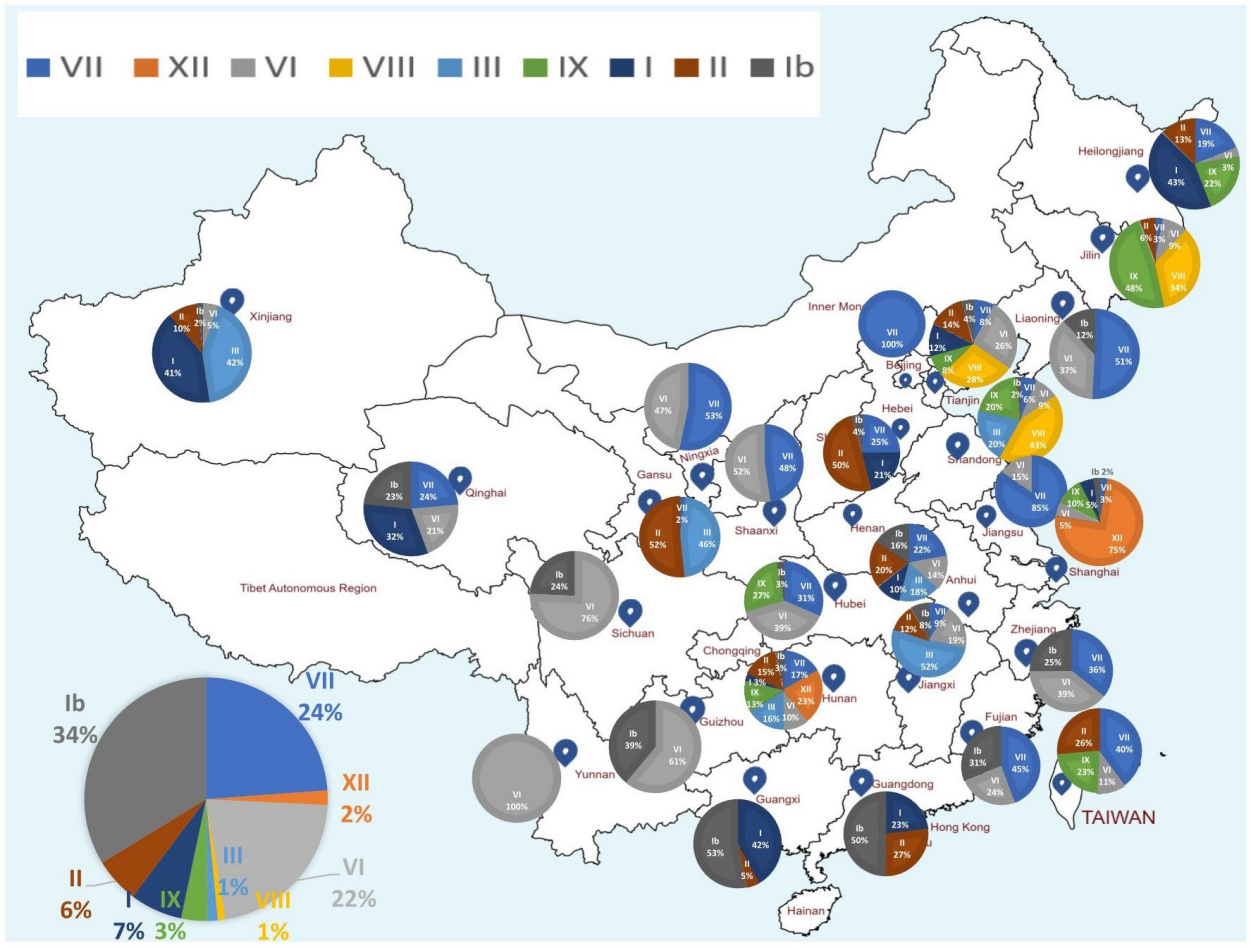
Map showing the geographical distribution of NDV genotypes in China based on the full-length genomes. The pie charts demonstrate the relative percentage of NDV genotypes for each of the 26 provinces. The pie charts in the left corner indicates the relative percentage of NDV genotypes included in this analysis. NDV genotypes are color coded. Provinces’ names and borders are shown. Ib genotype is found to dominate China (34%), followed by VII (24%) and VI (22%). Hunan province has the highest genotypic diversity with eight reported genotypes (VII, XII, VI, III, IX, I, II, and Ib).

The ML phylogenetic tree based on full-length genomes showed that NDV strains isolated in China during 1946–2020 from different species, including chicken and duck, pigeon, Rough-legged Buzzard, White-fronted Goose, Wild bird, and swine, cluster into two major clades, GI and GII, where GI encompasses a single sub-clade Ib, while GII comprises eight sub-clades (I, II, III, VI. VII. VIII, IX and XII; Figure 1; Supplementary Figure S1). The genotype Ib within GI clade, and VII and VI genotypes within GII clade revealed the highest number of strains, with *N* = 174, 123, and 112 strains, respectively, indicating that Ib genotype dominates China, particularly the Central and East China (Hunan = 61 and Jiangxi = 52 strains, respectively; Table 1; Figure 2). In contrast, the VIII genotype is identified to be uncommonly distributed in China with only 3 strains in Jiangsu: QH1(GenBank ID: FJ751918), QH4 (GenBank ID: FJ751919), and HR09 (GenBank ID: MF285077), one strain in Guangxi: GXGB2011 (GenBank ID: MH715892), and one in Shaanxi: Italien (GenBank ID: EU293914; Table 1; Supplementary Figure S1). Furthermore, the VI genotype is shown with a large geographical distribution and has been identified in twenty-one provinces, followed by VII in twenty provinces, and Ib in seventeen provinces. Hunan province (Central China), Heilongjiang (Northeast), and Jiangxi (East China) represent the geographical regions where most of the NDV strains were isolated (*N* = 72, 65, and 62, respectively), whereas Hunan province shows the greatest NDV diversity among the twenty-six provinces by having eight NDV genotypes (VII, XII, VI, III, IX, I, II, and Ib) except the VIII genotype (Table 1; Figure 2). The Ib genotype is found to dominate China (34%), particularly South and East China, followed by VII (24%) and VI (22%; Figure 2).

### 3.2. Genomic characteristics of the China NDV strains

**Figure 3:**
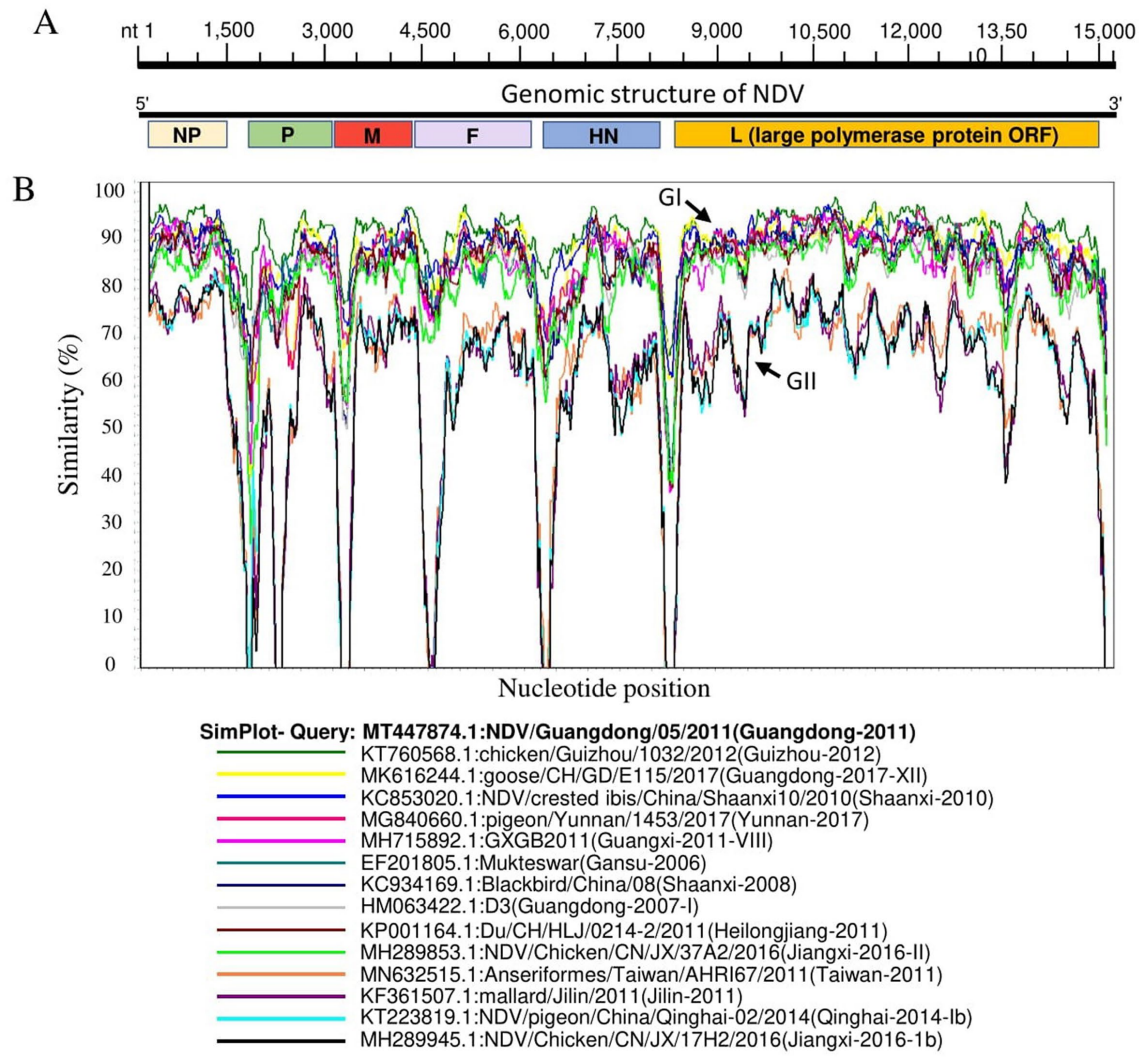
Similarity map of fifteen China NDV full-length representative strains. **(A)** Schematic diagram of the complete NDV genomic structure. From the 5′ end to the 3′ end are the ORFs encoding NP, P, M, F, HN, and L (large polymerase protein). **(B)** SimPlot similarity analysis results using SimPlot ver.3.5.1. The virus NDV/Guangdong/05/2011(GenBank ID:MT447874.1) identified in Guangdong province in 2011 was used as the query sequence to compare with fourteen other representative strains from China.

Genomic sequence comparison of NDV strains circulating in China revealed that F, HN, M and P gene sequences exhibit the lowest genetic similarity levels, while the L gene encoding large polymerase protein is shown to be the most stable with the greatest similarity levels (Figure 3). Consistent with the phylogenetic tree results, the full-length genomic sequences of the NDV strains collected in China fell separately into two distinct groups: GI and GII (Figure 3).

### 3.3. The phylogeographic network analysis of full-length genomes of China NDV, 1946–2020

**Figure 4:**
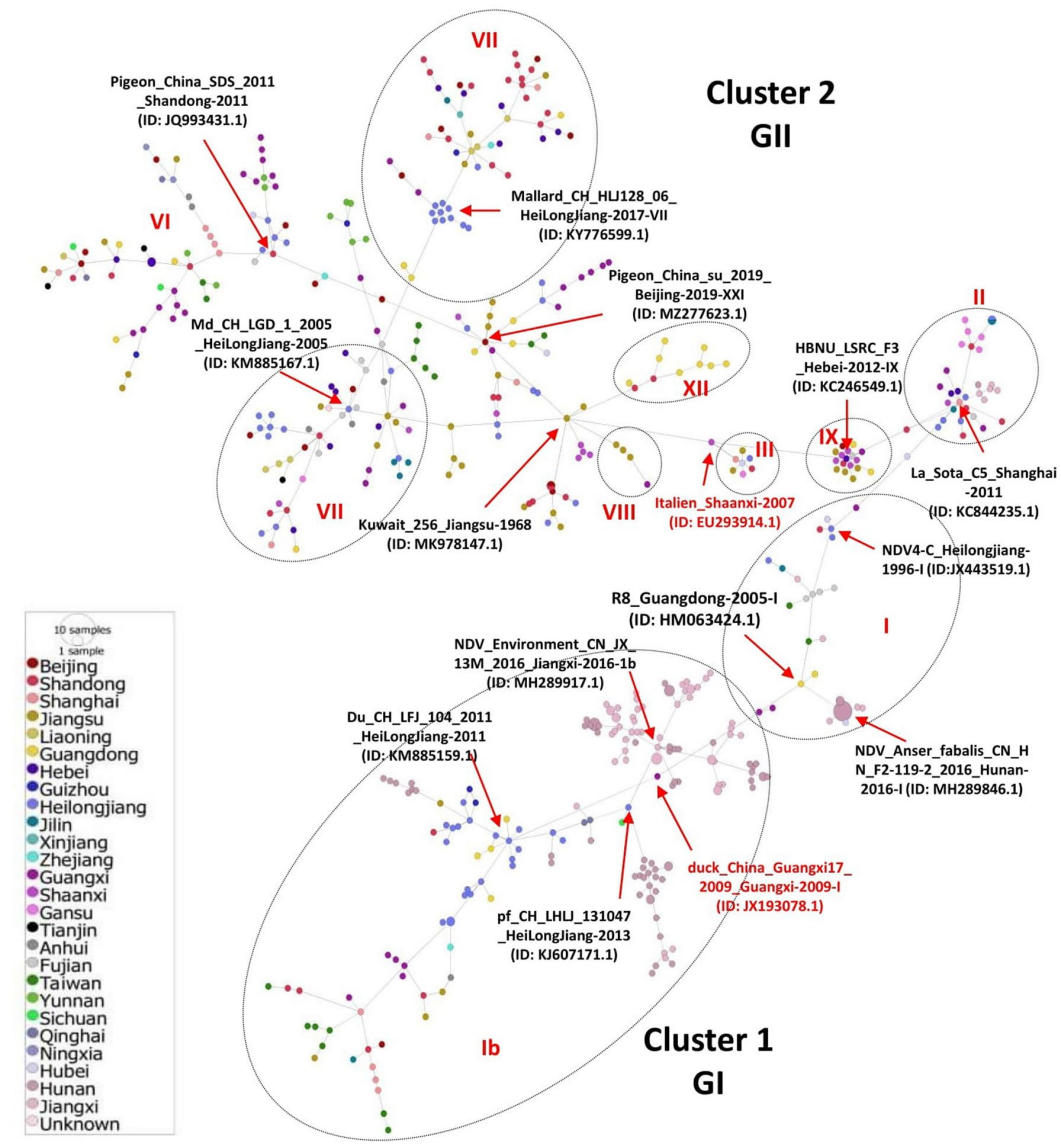
Phylogeographic network analysis of China NDV complete genomic sequences. The phylogenetic network of 517 full-length genomes of NDVs was inferred using the Minimum Spanning Network (MSN) implemented by PopArt v1.7. NDV strains collected from 26 provinces in China were grouped into two major Network Clusters corresponding to GI and GII groups, with further sub-branches. The distance is proportional to the number of mutations. Colors represent different provinces of China.

We inferred the phylogeographic network of all the 517 full-length NDV sequences to map the regional spread of NDVs during 1946–2020. In agreement with our ML phylogenetic tree, the phylogeographic analysis also showed two distinct mutational Network Clusters (Figure 4). The network analysis suggests that NDV strains in China are possibly radiated from strains reported in Hunan, especially the NDV strain Anser_fabalis_CN_HN_F2-119-2_2016_I (GenBank ID: MH289846.1) isolated in Hunan in 2016. The Network Cluster 1 (GI) is connected by four strains, e.g., R8_Guangdong-2005-I (GenBank ID: HM063424.1), CN_JX_13M_2016_Jiangxi-2016-1b (GenBank ID: MH289917.1), pf_CH_LHLJ_131047_HeiLongJiang-2013 (GenBank ID: KJ607171.1) and Du_CH_LFJ_104_2011_HeiLongJiang-2011 (GenBank ID: KM885159.1). On the other hand, cluster 2 (GII) was shown to have high diversity with multiple short and long mutational branches, where three strains, La_Sota_C5_Shanghai-2011 (GenBank ID: KC844235.1), Md_CH_LGD_1_2005_HeiLongJiang-2005 (GenBank ID: KM885167.1) and Pigeon_China_su_2019_Beijing-2019-XXI (GenBank ID: MZ277623.1) connect most of the branches (Figure 4).

### 3.4. Potential recombination among China NDV strains

Genetic recombination is known to be one of the main driving forces behind the viral evolution and genetic diversity of pathogens over time, impacting their virulence and Spatio-Temporal spread. Given the possible effects of recombination on the security of NDV as an oncolytic virus and attenuated vaccine, we attempted screening genetic exchanges and recombination patterns among our dataset (whole genome sequences of 517 China NDVs). We identified 34 potential recombination events between 1946 and 2020, of which 17 were intragenotype and 15 were intergenotype, while only two occurred between GI/GII clades: Events 1 and 4 (type Ib vs. I; Table 2). Five recombination events occurred between strains of the GI clade (Ib vs. Ib, Events 14, 16, 17, 18, 25); meanwhile, 27 other events occurred between strains of the GII clade, twelve of which were intragenotype (Events 20, 23, 24, and 26–34), and fifteen intergenotype (Events 2, 3, 5–13, 15, 19, 21, and 22). Importantly, our findings indicate that genotype VII is the most frequently involved in potential recombination. Further, we have observed that potential recombination involved strains from different species, dominated by chicken and duck, in addition to pigeon, Rough-legged Buzzard, White-fronted Goose, Wild bird, and swine (Table 2).

**Table 2:** Identification of 34 potential recombination events in the genome of NDVs using the RDP4 software package.

Recombination Event serial number	Recombinant		Minor parent		Major parent		Detection methods						
	GenBank ID: virus name (province-year)	Genotype	GenBank ID: virus name (province-year)	Genotype	GenBank ID: virus name (province-year)	Genotype	R	G	B	M	C	S	T
1	JX193078.1:duck/China/Guangxi17/2009(Guangxi-2009-I)	I	JN688863.1:Ch-ZJ-10-03(Zhejiang-2010-I)	Ib	JX193081.1:duck/China/Guangxi20/2010(Guangxi-2010-I)	I	+	+	+	+	+	−	+
2	GQ994433.1:XD/Shandong/08(Shandong-2008)	II	KC542895.1:Chicken/China/Hebei/01/2006(Hebei-2006)	VII	EU546165.2:JL-1(Jilin-2008-II)	II	+	+	+	+	−	+	+
3	GQ994433.1:XD/Shandong/08(Shandong-2008)	II	GQ994434.1:QG/Hebei/07(Hebei-2007)	VII	KM885165.1:Du/CH/LAH/209/2011(HeiLongJiang-2011)	II	+	+	+	+	+	+	+
4	JX193078.1:duck/China/Guangxi17/2009(Guangxi-2009-I)	I	KM885153.1:Du/CH/LJS/016/2011(HeiLongJiang-2011)	Ib	JX193077.1:duck/China/Guangxi16/2008(Guangxi-2008-I)	I	+	+	+	+	+	+	+
5	KF219497.1:duck/CH/GD/NH/10(Guangdong-2010-IX)	IX	KF219498.1:duck/CH/GD/SS/10(Guangdong-2010-VII)	VII	FJ436306.1:JS/1/02/Du(Jiangsu-2008-IX)	IX	+	+	+	+	+	+	+
6	AY225110.1:V4(Hubei-2003)	I	KR338979.1:chicken/China/SD0901/2009(Shandong-2009)	I	EU546165.2:JL-1(Jilin-2008-II)	II	+	+	+	+	+	+	+
7	GQ994434.1:QG/Hebei/07(Hebei-2007)	VII	KR338979.1:chicken/China/SD0901/2009(Shandong-2009)	I	KX765179.1:DU-FJ-241(Fujian-2014)	VII	+	+	+	+	+	+	+
8	EU167540.1:SRZ03(Beijing-2007)	VII	DQ060053.1:AQI-ND026(Shandong-2005)	II	KX765179.1:DU-FJ-241(Fujian-2014)	VII	+	+	+	+	+	+	+
9	AY225110.1:V4(Hubei-2003)	I	KR338979.1:chicken/China/SD0901/2009(Shandong-2009)	I	KM885165.1:Du/CH/LAH/209/2011(HeiLongJiang-2011)	II	+	+	+	+	+	+	+
10	DQ485230.1:chicken/China/Guangxi9/2003(Guangxi-2006)	VII	KY247177.1:SH15(Shanghai-2015)	III	KX765177.1:DU-FJCL117(Fujian-2011)	VII	+	+	−	+	+	+	+
11	JF930146.1:B/XJ/9/2009(Shandong-2009-IX)	IX	AY225110.1:V4(Hubei-2003)	I	KF219497.1:duck/CH/GD/NH/10(Guangdong-2010-IX)	IX	+	+	+	+	+	+	+
12	JF930146.1:B/XJ/9/2009(Shandong-2009-IX)	IX	AY845400.2:LaSota(HeiLongJiang-2004)	II	KF219497.1:duck/CH/GD/NH/10(Guangdong-2010-IX)	IX	+	+	+	+	+	+	+
13	JF930146.1:B/XJ/9/2009(Shandong-2009-IX)	IX	AY845400.2:LaSota(HeiLongJiang-2004)	II	FJ436306.1:JS/1/02/Du(Jiangsu-2008-IX)	IX	+	+	+	+	+	+	+
14	JN688863.1:Ch-ZJ-10-03(Zhejiang-2010-I)	Ib	JN688864.1:D-AH-10-02(Anhui-2010-I)	Ib	MF100763.1:D/GD/GZ/817/2016(Guangdong-2016-I)	Ib	+	+	+	+	+	+	+
15	JX193076.1:chicken/China/Guangxi15/2010(Guangxi-2010-VIId)	VII	KY776604.1:Mallard/CH/HLJ383/06(HeiLongJiang-2017-III)	III	DQ486859.1:GM(Guangdong-2006)	VII	+	+	+	+	+	+	+
16	KY776595.1:LTL130830(HeiLongJiang-2017-I)	Ib	JN688864.1:D-AH-10-02(Anhui-2010-I)	Ib	KJ499462.1:sw/CH/LHLJ/120608(HeiLongJiang-2012)	Ib	+	−	+	+	+	−	+
17	JN688863.1:Ch-ZJ-10-03(Zhejiang-2010-I)	Ib	JN688864.1:D-AH-10-02(Anhui-2010-I)	Ib	KM885153.1:Du/CH/LJS/016/2011(HeiLongJiang-2011)	Ib	+	+	−	−	−	+	+
18	*FJ794269.1:DV08-004(Shangdong-2008-I)	Ib	JQ713944.1:9a5b-D5C1(Shanghai-2009)	Ib	JN688864.1:D-AH-10-02(Anhui-2010-I)	Ib	+	+	−	+	+	+	+
19	JX193078.1:duck/China/Guangxi17/2009(Guangxi-2009-I)	I	JX193080.1:duck/China/Guangxi19/2009(Guangxi-2009-II)	II	MH289831.1:NDV/Duck/CN/JX/75C2/2016(Jiangxi-2016-I)	I	+	+	+	+	+	+	+
20	JX193083.1:duck/China/Guangxi22/2010(Guangxi-2010-I)	II	JX193081.1:duck/China/Guangxi20/2010(Guangxi-2010-I)	I	MH289831.1:NDV/Duck/CN/JX/75C2/2016(Jiangxi-2016-I)	I	+	+	+	+	+	+	+
21	JX193083.1:duck/China/Guangxi22/2010(Guangxi-2010-I)	II	JX193081.1:duck/China/Guangxi20/2010(Guangxi-2010-I)	I	JX193080.1:duck/China/Guangxi19/2009(Guangxi-2009-II)	II	+	+	+	+	+	+	+
22	JX193083.1:duck/China/Guangxi22/2010(Guangxi-2010-I)	II	JX193081.1:duck/China/Guangxi20/2010(Guangxi-2010-I)	I	JF950510.1:LaSota(Shangdong-2010)	II	+	+	+	+	+	−	+
23	JX193075.1:chicken/China/Guangxi14/2002(Guangxi-2002-VIId)	VII	JX193076.1:chicken/China/Guangxi15/2010(Guangxi-2010-VIId)	VII	KJ782375.1:go/CH/GD-QY/1997(HeiLongJiang-1997)	VII	+	+	+	+	+	+	+
24	KY776593.1:Buzzard/CH/HLJ013/06(HeiLongJiang-2017-VII)	VII	KY776592.1:Buzzard/CH/HLJ09/06(HeiLongJiang-2017-VII)	VII	KY776610.1:WfG/CH/HLJ052/06(HeiLongJiang-2017-VII)	VII	+	+	−	+	+	−	+
25	*KM885163.1:Du/CH/LFJ/073/2013(HeiLongJiang-2013)	Ib	KM885160.1:Du/CH/LFJ/027/2011(HeiLongJiang-2011)	Ib	KY776595.1:LTL130830(HeiLongJiang-2017-I)	Ib	+	+	+	+	+	−	+
26	MT301959.1:Goose/GD/F424/2019(Guangdong-2019-XII)	XII	MT301960.1:Goose/GD/F425/2019(Guangdong-2019-XII)	XII	MT301957.1:Goose/GD/F401/2019(Guangdong-2019-XII)	XII	+	+	−	+	+	+	+
27	JX193074.1:egret/China/Guangxi/2011(Guangxi-2011-VIIa)	VII	JX193075.1:chicken/China/Guangxi14/2002(Guangxi-2002-VIId)	VII	KX765879.1:chicken/Yunnan/1027/2016(Yunnan-2016)	VII	+	−	+	−	−	+	+
28	*MT301959.1:Goose/GD/F424/2019(Guangdong-2019-XII)	XII	MT301960.1:Goose/GD/F425/2019(Guangdong-2019-XII)	XII	MT301957.1:Goose/GD/F401/2019(Guangdong-2019-XII)	XII	+	+	−	+	+	+	+
29	MN862505.1:Pigeon/Shandong/3P3/2018(Shandong-2018)	VI	MN862497.1:Pigeon/Beijing/BJ96/1996(Beijing-1996)	VI	MK749297.1:Pi/CH/GXG1/2012(Guangxi-2012)	VI	+	+	−	+	+	+	+
30	KY776608.1:WB/CH/HLJ001/06(HeiLongJiang-2017-VII)	VII	KY776592.1:Buzzard/CH/HLJ09/06(HeiLongJiang-2017-VII)	VII	KY776593.1:Buzzard/CH/HLJ013/06(HeiLongJiang-2017-VII)	VII	+	+	−	+	+	+	+
31	GQ994434.1:QG/Hebei/07(Hebei-2007)	VII	KC542913.1:Chicken/China/Shandong/02/2012(Shandong-2012)	VII	EU167540.1:SRZ03(Beijing-2007)	VII	+	+	+	+	+	+	+
32	KY776601.1:Mallard/CH/HLJ361/05(HeiLongJiang-2017-VII)	VII	KY776602.1:Mallard/CH/HLJ363/05(HeiLongJiang-2017-VII)	VII	KY776593.1:Buzzard/CH/HLJ013/06(HeiLongJiang-2017-VII)	VII	+	+	−	+	+	+	+
33	KJ600786.1:Du/CH/SD/2009/134(Shanghai-2009-VII)	VII	KJ600785.1:Ch/CH/SD/2008/128(Shanghai-2008-VII)	VII	GQ994434.1:QG/Hebei/07(Hebei-2007)	VII	+	+	+	+	+	+	+
34	KJ600786.1:Du/CH/SD/2009/134(Shanghai-2009-VII)	VII	KJ600785.1:Ch/CH/SD/2008/128(Shanghai-2008-VII)	VII	GQ994434.1:QG/Hebei/07(Hebei-2007)	VII	+	+	−	+	+	+	+

R, RDP; G, GENECONV; B, BootScan; M, MaxChi; C, Chimera; S, SiScan; T, 3seq; +, verified; −, not verified.

* The major or minor parent may be the actual recombinant due to the possibility of misidentification.

The potential recombination events were identified by each of the seven algorithms (RDP, GENECONV, Bootscan, MaxChi, Chimera, SiScan, and 3seq) embedded in the RDP4 package.

**Figure 5:**
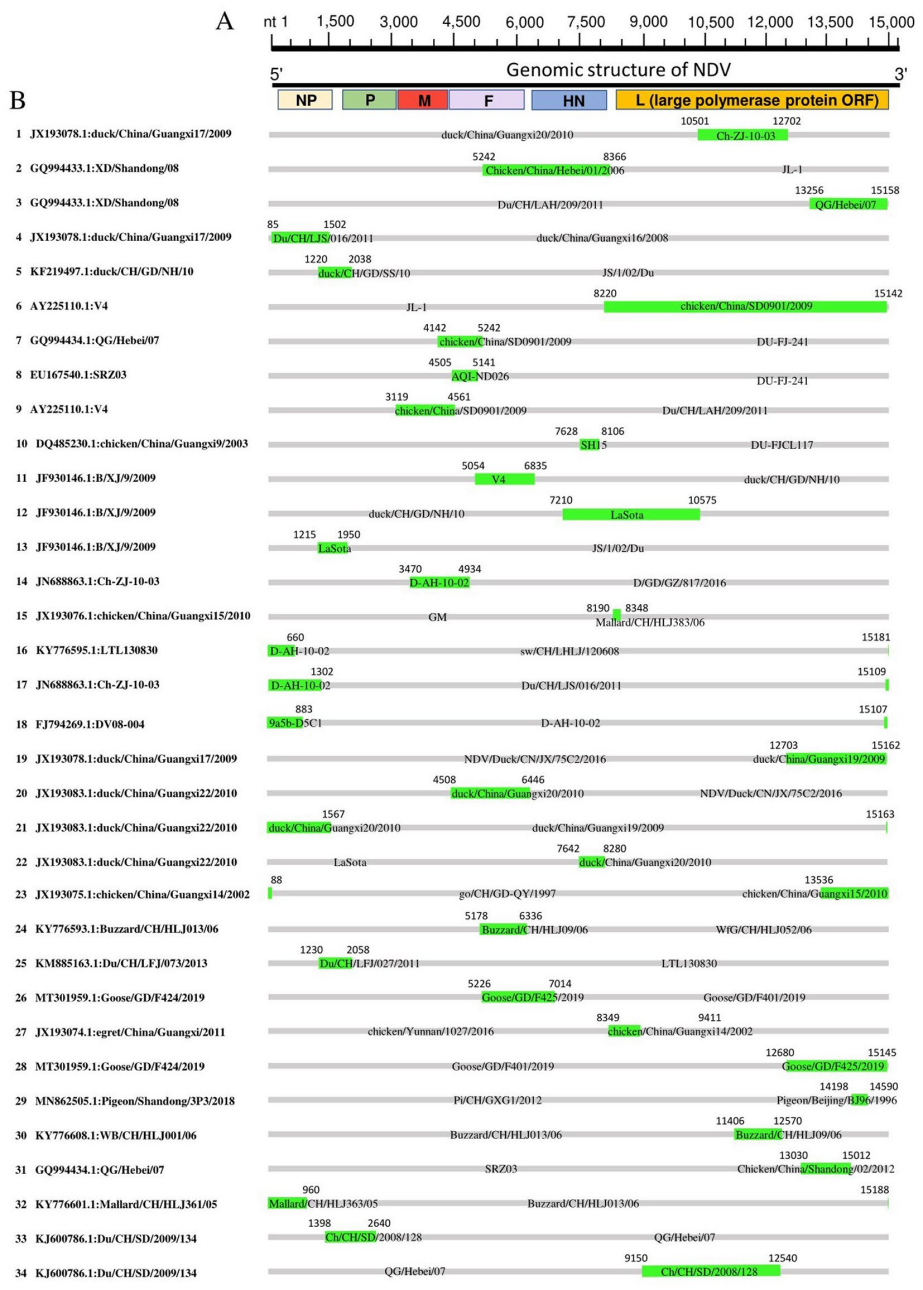
Recombination map of NDV full-length genomic sequences (Event 1 to Event 34). **(A)** The schematic of the complete NDV genomic structure is shown at the top. **(B)** The genome map of the recombinant NDVs is cited in Table 2. The grey blocks correspond to the major parents, and the green blocks correspond to the minor parents. Numbers on the blocks indicate the beginning and ending breakpoints of the recombination. The serial numbers of the recombination events are on the left, and recombinants are cited in the format of GenBank ID: virus name.

We mapped the breakpoints of the identified recombination events across the complete genomes and observed that ten of them occurred within the large polymerase protein ORF (L protein), including Events 1, 3, 5, 19, 23, 28, 29, 30, 31, 34 (Figure 5), six within the NP protein (Events 4, 16, 17, 18, 21, 32), four within the F protein (Events 7, 8, 20, 24), one within M protein (Event 9) and one involved both M and F coding regions (Event 14; Figure 5). Therefore, the L protein appears to be a hotspot for the genetic exchanges in China NDV genomes.

Further, we substantiated the putative recombinant events by generating phylogenetic trees based on different genomic fragments. As shown in Supplementary Figures S2, S3, the phylogenetic trees are not superimposed. In Event 3, the recombinant GQ994433 in the fragment nt 13,000–15,000 based phylogenetic tree is genetically closer to the minor parent QG/Hebei07 (Supplementary Figure S2A). However, in the fragment nt 5,100–6,400-based phylogenetic tree, the recombinant GQ994433 is genetically closer to the major parent NDV/Duck/CN/JX/75C2/ 2016 (Supplementary Figure S3A). The analysis of other recombination events revealed similar results, confirming the authenticity of the identified recombinants. Furthermore, Simplot genomic similarity analysis using corresponding recombinant as a query also corroborated the recombination events (Supplementary Figure S4).

### 3.5. Amino acids variability of NDV proteins

**Figure 6:**
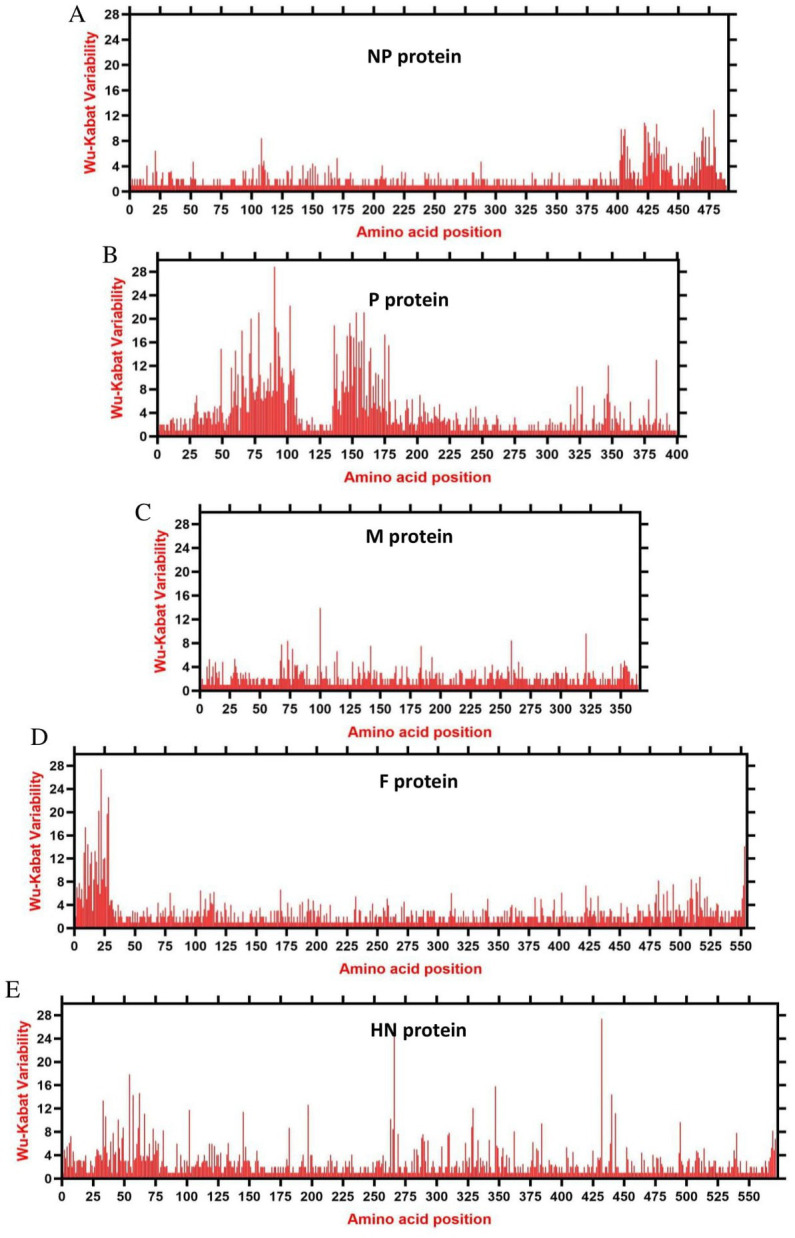
The landscape of amino acid variation in China NDV strains during 1946–2020 determined by Wu-Kabat amino acids variability plotting. **(A)** NP protein, **(B)** P protein, **(C)** M protein, **(D)** F protein, and **(E)** HN protein. Y-axes indicate the Wu-Kabat variability coefficient values, where the estimation limit was “1.” Above the threshold of “> 1” are variations. X-axes indicate the amino acid positions relative to the corresponding proteins.

**Figure 7:**
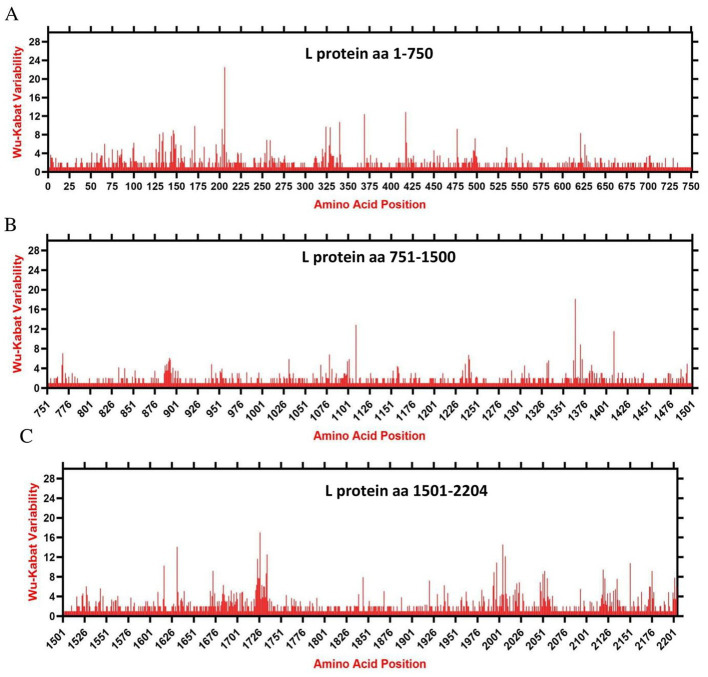
The landscape of amino acid variation in the L protein of China NDV strains during 1946–2020 determined by Wu-Kabat amino acids variability plotting. **(A)** Fragment aa 1-750, **(B)** Fragment aa 751-1,500, and **(C)** Fragment aa 1,501-2,204. Y-axes indicate the Wu-Kabat variability coefficient values, where the estimation limit was “1.” Above the threshold of “> 1” are variations. X-axes indicate the amino acid positions relative to the corresponding proteins.

We assessed the amino acids variability landscape by implementing the Wu-Kabat variability coefficient, provided by the Protein Variability Server (PVS), and acquired the consensus sequences of the NDV ORFs encoding the NP, P, M, F, HN, and L proteins. The consensus sequence of NP protein was 489 aa, P protein 399 aa, M 364aa, F 553 aa, and HN protein was 571 aa long (Figures 6A–E), whereas the consensus sequences of L protein were shown in Figures 7A–C. The P protein was identified to be the most variable protein of NDV (Figure 6B), followed by the HN (Figure 6E), F protein (Figure 6D) and NP protein (Figure 6A), while the M protein (Figure 6C) was found to be relatively conserved. Fragment aa 1,501–2,204 (Figure 7C) is identified as highly variable compared to fragment aa 1–750 (Figure 7A) and fragment aa 751–1,500 (Figure 7B) of the L protein.

## 4. Discussion

Recombination is an important evolutionary event of pathogens that continuously shapes virus population variability; however, its role in the evolution of negative-sense RNA viruses, particularly NDV, has been debated. Herein, we assessed the evolution diversities among 517 complete genome sequences of China NDV strains. Although recombination in negative-sense genome RNA viruses has been reported to occur at a low rate (46), in this study, we identified a great number of recombination events occurring among China NDVs’ complete genome sequences (34/517). Further, we have observed that NDV potential recombination might occur between strains from different species, particularly (chicken vs. duck), and provided evidence that potential recombination also may occur between NDV strains of swine and chicken lineages. Multiple potential recombinants were identified to be resulting from different parental strains, such as recombinant JX193078 (Events 1, 4, 19), recombinant GQ994433 (Events 2 and 3), recombinant AY225110 (Events 6 and 9), recombinant, JF930146 (Events 11, 12, and 13) X193083 (Events 20, 21, and 22), recombinant MT301959 (Events 26 and 28), and recombinant KJ600786 (Events 30 and 34). The aforementioned identified potential recombinants result from at least three different parental sequences, which might indicate the high susceptibility of some NDV strains to recombine with more viruses, thus generating more new lineages. However, the recombination events identified in this report were resulted from the bioinformatic analysis without further confirmation by successful isolation of the recombinant viruses from the single plaque clonal seletion, thus, the existence of possibility of sequencing-based artificial recombination cannot be completely exluded.

Given the substantial economic losses to the industry, intensive vaccination against NDV is the main adopted control strategy (47). However, the available inactivated and live attenuated NDV vaccines are reported to decrease and prevent clinical ND but are unable to reduce the virus shedding (48). Consistently, our results have identified that potential recombination among China NDV involves vaccine strains and occurs between circulating and vaccine NDV viruses. The recombinant JX193083 (Event 22) is found to be resulting from recombination between the attenuated vaccine strain LaSota (JF950510.1), the most widely applied vaccine (49), and a circulating strain duck/China/Guangxi20/ 2010 (JX193081) as major and minor parental sequences, respectively. The same recombinant JX193083 is found resulting from different parental strains in Event 20 and Event 21. Furthermore, the live attenuated vaccine strain “V4” (AY225110) (49) is found to be involved in the recombinant Event 11 as a minor parental sequence, and itself is a recombinant in Event 6 resulting from recombination between chicken/China/ SD0901/2009 (KR338979) and JL-1(EU546165.2), and in Event 9 resulting between chicken/ China/ SD0901/2009 (KR338979.1) and Du/CH/LAH/209/2011(KM885165.1) as minor and major parental sequences, respectively. Therefore, we provided evidence that NDV potential recombination between China NDV circulating viruses and NDV vaccines may occur and suggest that immunity imposed by the applied vaccination might be the driving force of NDV recombination and one of the reasons behind the NDV’s continuous evolution.

To better understand the epidemiology of China NDV strains, we generated a phylogenetic tree based on the full-length genomes of 517 NDV strains and have found that China strains cluster into two main groups GI comprising a single genotype and GII group subdivided into eight further genotypes. Previously, it has been reported based on the F gene phylogenetic tree of NDV that the VII genotype is prevalent in China and that VIIc and VIId subgenotypes exist in China (50–52). Our report identified genotype Ib as the most dominant, followed by VII and VI. Viral adaptation to new environments and new hosts depends highly on the ability to rapidly generate genetic diversity (53), and the evolution of prevalent genotypes tends to be influenced by vaccine immune pressure (54). Therefore, these findings need more experimental exploration to deeply explain the mechanisms.

Here, Shandong province in East China shows the greatest NDV diversity, while Hunan province, Heilongjiang, and Jiangxi represent the geographical regions where most NDV strains were reported. The XII genotype comprising virulent viruses is identified as emerging in Guangdong and is found to encompass recombinant strains: Event 28 (MT301959) collected in 2019 and resulting from recombination between a major (MT301957) and a minor (MT301960) parental sequences collected in 2019 alarming new lineages. The largest economic circles of China, the Yangtze River Delta located in the East and the Pearl River Delta in the South (55), are regions of the highest poultry population and farming and transportation network, which might explain in part the distribution and genetic exchanges among NDV strains. Further, we reconstructed the China NDV infection paths using the phylogeographic network analysis to the evolutionary scenario. NDV strains follow two major Network Clusters, with a possible ancestral node from Hunan: strain CN_HN_F2-119-2_2016_I (MH289846.1), corroborating the phylogenetic tree results.

Recently, oncolytic virotherapy has been known to have tremendous advances in recent years. As a virotherapeutic agent, NDV has demonstrated efficacious oncolytic activities in both preclinical and clinical studies (30–32). The previous phylogenetic analysis has reported that the virulent and oncolytic NDV Italien strain (EU293914), cytotoxic to tumor cells within 24 h, is classified in the IV genotype (56). However, the EU293914 Italien strain seems to be independent within genotype III of the GII group, genetically close to the NDV vaccine Mukteswar strain (JF950509). Therefore, our results revealed that NDV genomes may not be stable as it was thought. Our report findings suggest that with extensive recombination of the NDV genetic material, the risk of generating strains with an unstable genome exists and that during replication of NDV within tumor cells as an oncolytic virus might produce new, less efficient progeny or might revert to the wild form. The identified recombinants, especially genetic exchanges within F and HN coding regions together, might complicate the virulence and complicate research studies on both the oncolytic effect of NDVs and the vaccine design against NDVs.

In summary, it has to be considered that avian co-infection with NDV vaccine strains and NDV circulating strains may influence viral virulence and the emergence of NDV outbreaks. Therefore, it is necessary to thoroughly review the relationship between NDV vaccination and circulating strains.

## Data availability statement

The original contributions presented in the study are included in the article/Supplementary material, further inquiries can be directed to the corresponding author.

## Author contributions

AB and LX: conceptualization. PS, AB, P-HW, and Y-YG: data analysis. AB and J-QZ: visualization and writing. CW and LX: administration. AB, PS, and LX: manuscript revision. All authors contributed to the article and approved the submitted version.

## Funding

This work was supported by the Program of Introducing Talents of Discipline to Universities (D21004).

## Conflict of interest

The authors declare that the research was conducted in the absence of any commercial or financial relationships that could be construed as a potential conflict of interest.

## Publisher’s note

All claims expressed in this article are solely those of the authors and do not necessarily represent those of their affiliated organizations, or those of the publisher, the editors and the reviewers. Any product that may be evaluated in this article, or claim that may be made by its manufacturer, is not guaranteed or endorsed by the publisher.

## Footnotes

^1^ https://ictv.global/taxonomyInternational

## References

[1] Buijs PR Verhagen JH van Eijck CH van den Hoogen BG. Oncolytic viruses: From bench to bedside with a focus on safety. Hum Vaccin Immunother. (2015) 11:1573–84. doi: 10.1080/21645515.2015.1037058, PMID: 25996182 PMC4514197

[2] Kaleta EF Baldauf C. Newcastle Disease in Free-Living and Pet Birds. Berlin: Springer (1988).

[3] Liu H Wang Z. *The history and current status of newcastle disease*. China Animal Health Inspection. (2015).

[4] Amarasinghe GK Ayllón MA Bào Y Basler CF Bavari S Blasdell KR. Taxonomy of the order Mononegavirales: update 2019. Arch Virol. (2019) 164:1967–80. doi: 10.1007/s00705-019-04247-4, PMID: 31089958 PMC6641539

[5] Nagai Y Hamaguchi M Toyoda T. Molecular biology of Newcastle disease virus. Prog Vet Microbiol Immunol. (1989) 5:16–64. 2696520

[6] Krishnamurthy S Samal SKJJOGV. Nucleotide sequences of the trailer, nucleocapsid protein gene and intergenic regions of Newcastle disease virus strain Beaudette C and completion of the entire genome sequence. J Gen Virol. (1998) 79:2419–24. doi: 10.1099/0022-1317-79-10-2419 9780047

[7] Alexander DJ. Newcastle disease and other avian paramyxoviruses. Rev Sci Tech. (2000) 19:443–62. doi: 10.20506/rst.19.2.1231 10935273

[8] Fields BN. Paramyxoviridae: the viruses and their replication In: Fields BN Knipe DN Howley PM, editors. Fields virology. New York: Lippincott, Williams, and Wilkins (2007)

[9] Steward M Vipond IB Millar NS Emmerson PT. RNA editing in Newcastle disease virus. J Gen Virol. (1993) 74:2539–47. doi: 10.1099/0022-1317-74-12-2539, PMID: 8277263

[10] Ganar K Das M Sughandha S Kumar S. Newcastle disease virus: Current status and our understanding. Virus Res. (2014) 466-467:1–158. doi: 10.1016/j.virusres.2014.02.016, PMID: 24589707 PMC7127793

[11] Deng R Wang Z Mirza AM Iorio RM. Localization of a domain on the paramyxovirus attachment protein required for the promotion of cellular fusion by its homologous fusion protein spike. Virology. (1995) 209:457–69. doi: 10.1006/viro.1995.1278, PMID: 7778280

[12] Melanson VR Iorio RM. Amino acid substitutions in the F-specific domain in the stalk of the newcastle disease virus HN protein modulate fusion and interfere with its interaction with the F protein. J Virol. (2004) 78:13053–61. doi: 10.1128/jvi.78.23.13053-13061.2004, PMID: 15542657 PMC525001

[13] Connaris H Takimoto T Russell R Crennell S Moustafa I Portner A. Probing the sialic acid binding site of the hemagglutinin-neuraminidase of Newcastle disease virus: identification of key amino acids involved in cell binding, catalysis, and fusion. J Virol. (2002) 76:1816–24. doi: 10.1128/jvi.76.4.1816-1824.2002, PMID: 11799177 PMC135884

[14] Panda A Huang Z Elankumaran S Rockemann DD Samal SK. Role of fusion protein cleavage site in the virulence of Newcastle disease virus. Microb Pathog. (2004) 36:1–10. doi: 10.1016/j.micpath.2003.07.003, PMID: 14643634 PMC7125746

[15] Romer-Oberdorfer A. Contribution of the length of the HN protein and the sequence of the F protein cleavage site to Newcastle disease virus pathogenicity. J Gen Virol. (2003) 84:3121–9. doi: 10.1099/vir.0.19416-0 14573818

[16] Huang Z Panda A Elankumaran S Govindarajan D Rockemann DD Samal SK. The hemagglutinin-neuraminidase protein of Newcastle disease virus determines tropism and virulence. J Virol. (2004) 78:4176–84. doi: 10.1128/jvi.78.8.4176-4184.2004, PMID: 15047833 PMC374304

[17] Jin J Zhao J Ren Y Zhong Q Zhang G. Contribution of HN protein length diversity to Newcastle disease virus virulence, replication and biological activities. Sci Rep. (2016) 6:36890. doi: 10.1038/srep36890, PMID: 27833149 PMC5105081

[18] Mebatsion T Weiland F Conzelmann KK. Matrix protein of rabies virus is responsible for the assembly and budding of bullet-shaped particles and interacts with the transmembrane spike glycoprotein G. J Virol. (1999) 73:242–50. doi: 10.1128/JVI.73.1.242-250.1999, PMID: 9847327 PMC103828

[19] Peeples ME Bratt MA. Mutation in the matrix protein of Newcastle disease virus can result in decreased fusion glycoprotein incorporation into particles and decreased infectivity. J Virol. (1984) 51:81–90. doi: 10.1128/jvi.51.1.81-90.1984, PMID: 6547186 PMC254403

[20] Dortmans JCFM Rottier PJM Koch G Peeters BPH. The Viral Replication Complex Is Associated with the Virulence of Newcastle Disease Virus. J Virol. (2010) 84:10113–20. doi: 10.1128/JVI.00097-10, PMID: 20660202 PMC2937822

[21] Rout SN Samal SK. The Large Polymerase Protein Is Associated with the Virulence of Newcastle Disease Virus. J Virol. (2008) 82:7828–36. doi: 10.1128/JVI.00578-08, PMID: 18550657 PMC2519556

[22] Cattoli G Susta L Terregino C Brown C. Newcastle disease: a review of field recognition and current methods of laboratory detection. J Vet Diagn Investig. (2011) 23:637–56. doi: 10.1177/1040638711407887 21908305

[23] Alexander DJ Aldous EW Fuller CMJ. The long view: a selective review of 40 years of Newcastle disease research. Avian Pathol. (2012) 41:329–35. doi: 10.1080/03079457.2012.697991, PMID: 22834545

[24] Czeglédi A Ujvári D Somogyi E Wehmann E Werner O Lomniczi B. Third genome size category of avian paramyxovirus serotype 1 (Newcastle disease virus) and evolutionary implications. Virus Res. (2006) 120:36–48. doi: 10.1016/j.virusres.2005.11.009, PMID: 16766077

[25] Dimitrov KM Ramey AM Qiu X Bahl J Afonso CL. Temporal, geographic, and host distribution of avian paramyxovirus 1 (Newcastle disease virus). Infect Genet Evol. (2016) 39:22–34. doi: 10.1016/j.meegid.2016.01.008, PMID: 26792710

[26] Snoeck CJ Owoade AA Couacy-Hymann E Alkali BR Okwen MP Adeyanju AT. High genetic diversity of Newcastle disease virus in poultry in west and central africa: cocirculation of genotype XIV and newly defined genotypes XVII and XVIII. J Clin Microbiol. (2013) 51:2250–60. doi: 10.1128/JCM.00684-13, PMID: 23658271 PMC3697698

[27] Du J Xia J Li S Shen Y Huang Y. Evolutionary dynamics and transmission patterns of Newcastle disease virus in China through Bayesian phylogeographical analysis. PLoS One. (2020) 15:e0239809. doi: 10.1371/journal.pone.0239809, PMID: 32991628 PMC7523974

[28] Zhang P Xie G Liu X Ai L Chen Y Meng X. High genetic diversity of Newcastle disease virus in wild and domestic birds in Northeastern China from 2013 to 2015 reveals potential epidemic trends. Appl Environ Microbiol. (2015) 82:1530–6. doi: 10.1128/AEM.03402-15 26712543 PMC4771317

[29] Cassel WA Garrett RE. Newcastle disease virus as an antineoplastic agent. Cancer. (2015) 18:863–8. doi: 10.1002/1097-0142(196507)18:7<863::aid-cncr2820180714>3.0.co;2-v 14308233

[30] Laurie SA Bell JC Atkins HL Roach J Bamat MK O'Neil JD. A phase 1 clinical study of intravenous administration of PV701, an oncolytic virus, using two-step desensitization. Clin Cancer Res. (2006) 12:2555–62. doi: 10.1158/1078-0432.Ccr-05-2038, PMID: 16638865

[31] Lorence RM Scot Roberts M O'Neil JD Groene WS Miller JA Mueller SN. Phase 1 clinical experience using intravenous administration of PV701, an oncolytic Newcastle disease virus. Curr Cancer Drug Targets. (2007) 7:157–67. doi: 10.2174/156800907780058853, PMID: 17346107

[32] Phuangsab A Lorence RM Reichard KW Peeples ME Walter RJ. Newcastle disease virus therapy of human tumor xenografts: antitumor effects of local or systemic administration. Cancer Lett. (2001) 172:27–36. doi: 10.1016/s0304-3835(01)00617-6 11595126

[33] Vijayakumar G Palese P Goff PH. Oncolytic Newcastle disease virus expressing a checkpoint inhibitor as a radioenhancing agent for murine melanoma. EBioMedicine. (2019) 49:96–105. doi: 10.1016/j.ebiom.2019.10.032, PMID: 31676387 PMC6945240

[34] Kim SH Samal SK. Newcastle disease virus as a vaccine vector for development of human and veterinary vaccines. Viruses. (2016) 8:183. doi: 10.3390/v8070183, PMID: 27384578 PMC4974518

[35] Russell L Peng KW. The emerging role of oncolytic virus therapy against cancer. Chin Clin Oncol. (2018) 7:16. doi: 10.21037/cco.2018.04.04, PMID: 29764161 PMC6557159

[36] Wei D Xu J Liu XY Chen ZN Bian H. Fighting Cancer with Viruses: Oncolytic Virus Therapy in China. Hum Gene Ther. (2018) 29:151–9. doi: 10.1089/hum.2017.212, PMID: 29284308

[37] Cuoco JA Rogers CM Mittal S. The oncolytic Newcastle disease virus as an effective immunotherapeutic strategy against glioblastoma. Neurosurg Focus. (2021) 50:E8. doi: 10.3171/2020.11.FOCUS20842, PMID: 33524945

[38] Huang F Dai C Zhang Y Zhao Y Wang Y Ru G. Development of Molecular Mechanisms and Their Application on Oncolytic Newcastle Disease Virus in Cancer Therapy. Front Mol Biosci. (2022) 9:889403. doi: 10.3389/fmolb.2022.889403, PMID: 35860357 PMC9289221

[39] Song H Zhong LP He J Huang Y Zhao YX. Application of Newcastle disease virus in the treatment of colorectal cancer. World J Clin Cases. (2019) 7:2143–54. doi: 10.12998/wjcc.v7.i16.2143, PMID: 31531310 PMC6718777

[40] Sievers F Wilm A Dineen D Gibson TJ Karplus K Li W. Fast, scalable generation of high-quality protein multiple sequence alignments using Clustal Omega. Mol Syst Biol. (2011) 7:539. doi: 10.1038/msb.2011.75, PMID: 21988835 PMC3261699

[41] Trifinopoulos J Nguyen L-T von Haeseler A Minh BQ. W-IQ-TREE: a fast online phylogenetic tool for maximum likelihood analysis. Nucleic Acids Res. (2016) 44:W232–5. doi: 10.1093/nar/gkw256, PMID: 27084950 PMC4987875

[42] Lole KS Bollinger RC Paranjape RS Gadkari D Kulkarni SS Novak NG. Full-length human immunodeficiency virus type 1 genomes from subtype C-infected seroconverters in India, with evidence of intersubtype recombination. J Virol. (1999) 73:152–60. doi: 10.1128/jvi.73.1.152-160.1999, PMID: 9847317 PMC103818

[43] Leigh JW Bryant D. POPART: full-feature software for haplotype network construction. Methods Ecol Evol. (2015) 6:1110–6. doi: 10.1111/2041-210X.12410

[44] Garcia-Boronat M Diez-Rivero CM Reinherz EL Reche PA. PVS: a web server for protein sequence variability analysis tuned to facilitate conserved epitope discovery. Nucleic Acids Res. (2008) 36:W35–41. doi: 10.1093/nar/gkn211, PMID: 18442995 PMC2447719

[45] Kabat E Wu TT Bilofsky H. Unusual distributions of amino acids in complementarity determining (hypervariable) segments of heavy and light chains of immunoglobulins and their possible roles in specificity of antibody-combining sites. J Biol Chem. (1977) 252:6609–16. doi: 10.1016/S0021-9258(17)39891-5, PMID: 408353

[46] Chare ER Gould EA Holmes EC. Phylogenetic analysis reveals a low rate of homologous recombination in negative-sense RNA viruses. J Gen Virol. (2003) 84:2691–703. doi: 10.1099/vir.0.19277-0, PMID: 13679603

[47] Bello MB Yusoff K Ideris A Hair-Bejo M Peeters BPH Omar AR. Diagnostic and vaccination approaches for Newcastle disease virus in poultry: the current and emerging perspectives. Biomed Res Int. (2018) 2018:7278459–18. doi: 10.1155/2018/7278459, PMID: 30175140 PMC6098882

[48] Roohani K Tan SW Yeap SK Ideris A Bejo MH Omar AR. Characterisation of genotype VII Newcastle disease virus (NDV) isolated from NDV vaccinated chickens, and the efficacy of LaSota and recombinant genotype VII vaccines against challenge with velogenic NDV. J Vet Sci. (2015) 16:447–57. doi: 10.4142/jvs.2015.16.4.447, PMID: 25643805 PMC4701737

[49] OIE. Newcastle disease. manual of standards for diagnostic tests and vaccines for terrestrial animals. In. Paris, France: World Organisation for Animal Health, (2008). 465–481.

[50] Qin ZM Tan LT Xu HY Ma BC Wang YL Yuan XY. Pathotypical characterization and molecular epidemiology of Newcastle disease virus isolates from different hosts in China from 1996 to 2005. J Clin Microbiol. (2008) 46:601–11. doi: 10.1128/jcm.01356-07, PMID: 18077643 PMC2238121

[51] Wang JY Liu WH Ren JJ Tang P Wu N Liu HJ. Complete genome sequence of a newly emerging newcastle disease virus. Genome Announc. (2013) 1:e00169. doi: 10.1128/genomeA.00196-13, PMID: 23661479 PMC3650438

[52] Wu S Wang W Yao C Wang X Hu S Cao J. Genetic diversity of Newcastle disease viruses isolated from domestic poultry species in Eastern China during 2005-2008. Arch Virol. (2011) 156:253–61. doi: 10.1007/s00705-010-0851-5, PMID: 21061026

[53] Sanjuán R Domingo-Calap P. Mechanisms of viral mutation. Cell Mol Life Sci. (2016) 73:4433–48. doi: 10.1007/s00018-016-2299-6, PMID: 27392606 PMC5075021

[54] Milani A Fusaro A Bonfante F Zamperin G Salviato A Mancin M. Vaccine immune pressure influences viral population complexity of avian influenza virus during infection. Vet Microbiol. (2017) 203:88–94. doi: 10.1016/j.vetmic.2017.02.016, PMID: 28619173

[55] Lu R. building engines for growth and competitiveness in China: experience with special economic zones and industrial clusters. Abingdon: Taylor & Francis (2011).

[56] Wei D Yang B Li YL Xue CF Chen ZN Bian H. Characterization of the genome sequence of an oncolytic Newcastle disease virus strain Italien. Virus Res. (2008) 135:312–9. doi: 10.1016/j.virusres.2008.03.003, PMID: 18420299

