# Supplementary material for: Multiple potential recombination events among Newcastle disease virus genomes in China between 1946 and 2020
Source: Front Vet Sci. 2023 May 3;10:1136855. doi: 10.3389/fvets.2023.1136855 (PMC10189042; doi:10.3389/fvets.2023.1136855)

Multiple Potential Recombination Events Among Newcastle Disease Virus (NDV) Genomes in China Between 1946-2020

Amina Nawal Bahoussi [1#], Pir Tariq Shah[1#], Jia-Qi Zhao[2], Pei-Hua Wang[1], Yan-Yan Guo[1], Changxin Wu[1,3,4,5], Li Xing[1, 3,4,5*]

Supplementary Figure S1 Phylogenetic analysis of China the Newcastle disease viruses. Phylogenetic relationship among 517 published NDV strains was based on the complete genomic nucleotide sequences collected between 1946-20 and obtained from the NCBI GenBank database and the tree construction was done using the ML method with the best-fitting model GTR+F+I+G4 in the IQ-TREE multicore version 1.6.12 with 1000 bootstrap replication. Viruses highlighted in red boxes were selected for Simplot genomic similarity analysis in Figure 3.

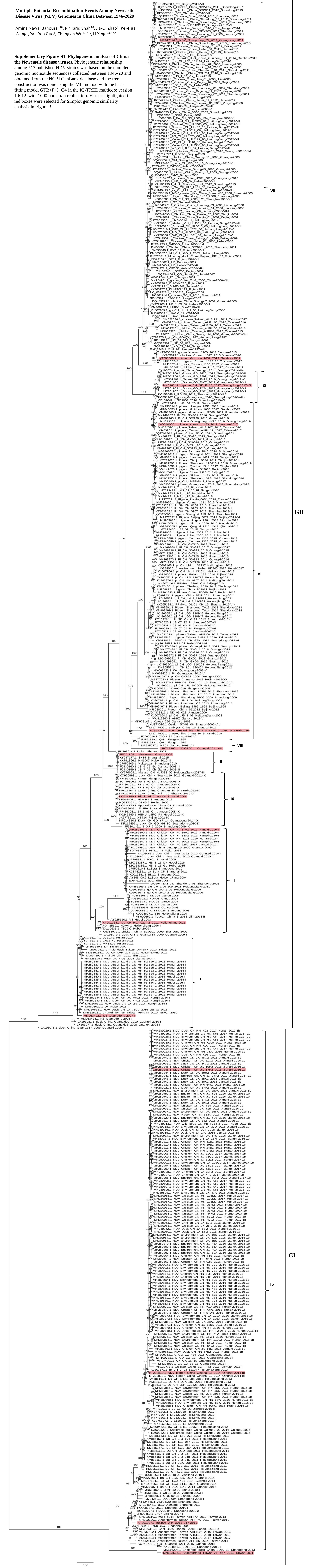

Supplement: Supplementary file 1 [file Data_Sheet_1.PDF]
