# Supplementary material for: Multiple potential recombination events among Newcastle disease virus genomes in China between 1946 and 2020
Source: Front Vet Sci. 2023 May 3;10:1136855. doi: 10.3389/fvets.2023.1136855 (PMC10189042; doi:10.3389/fvets.2023.1136855)
Supplement: Supplementary file 2 [file Data_Sheet_2.PDF]

Multiple Potential Recombination Events Among Newcastle Disease Virus (NDV) Genomes in China Between 1946-2020

Amina Nawal Bahoussi [1#], Pir Tariq Shah[1#], Jia-Qi Zhao[2], Pei-Hua Wang[1], Yan-Yan Guo[1], Changxin Wu[1,3,4,5], Li Xing[1, 3,4,5*]

Supplementary Figure S2 Phylogenetic analysis of NDV strains involved in the recombination events based on individual genomic fragments (Events 3, 4, 6, 12). **(A)** Genomic region nt 1- 1,300. **(B)** Genomic region nt 7,200-10,000**.** **(C)** Genomic region nt 13,000-15,000**.** Viruses are indicated in the format [virus name (GenBank ID: country-year of collection). The recombinant strains are labeled in red color. The minor and major parental sequences are labeled in blue and yellow, respectively. The numbers at each branch indicate the bootstrap values (%) of 1000 replicates. The scale bars indicate the number of inferred substitutions per site.

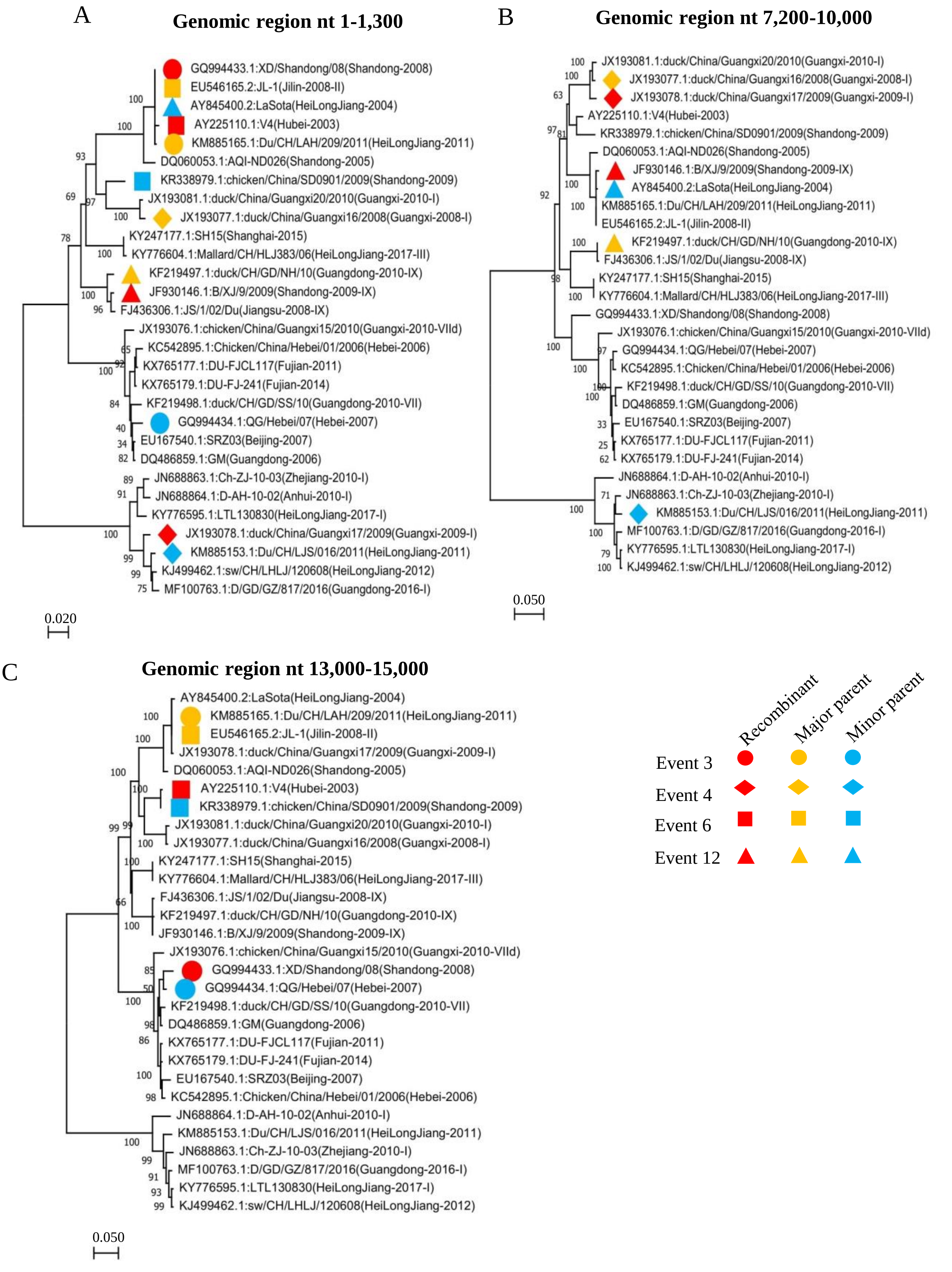

Supplementary Figure S3 Phylogenetic analysis of NDV viruses involved in the recombination events based on individual genomic fragments (Events 19, 23, 24, 28, 34). **(A)** Genomic region nt 5,100-6,400. **(B)** Genomic region nt 9,100-12,000. **(C)** Genomic region nt 13,000-15,000. Viruses are indicated in the format [virus name (GenBank ID: country-year of collection). The recombinant strains are labeled in red color. The minor parents and major parental sequences are labeled in blue and yellow, respectively. The numbers at each branch indicate the bootstrap values (%) of 1000 replicates. The scale bars indicate the number of inferred substitutions per site.

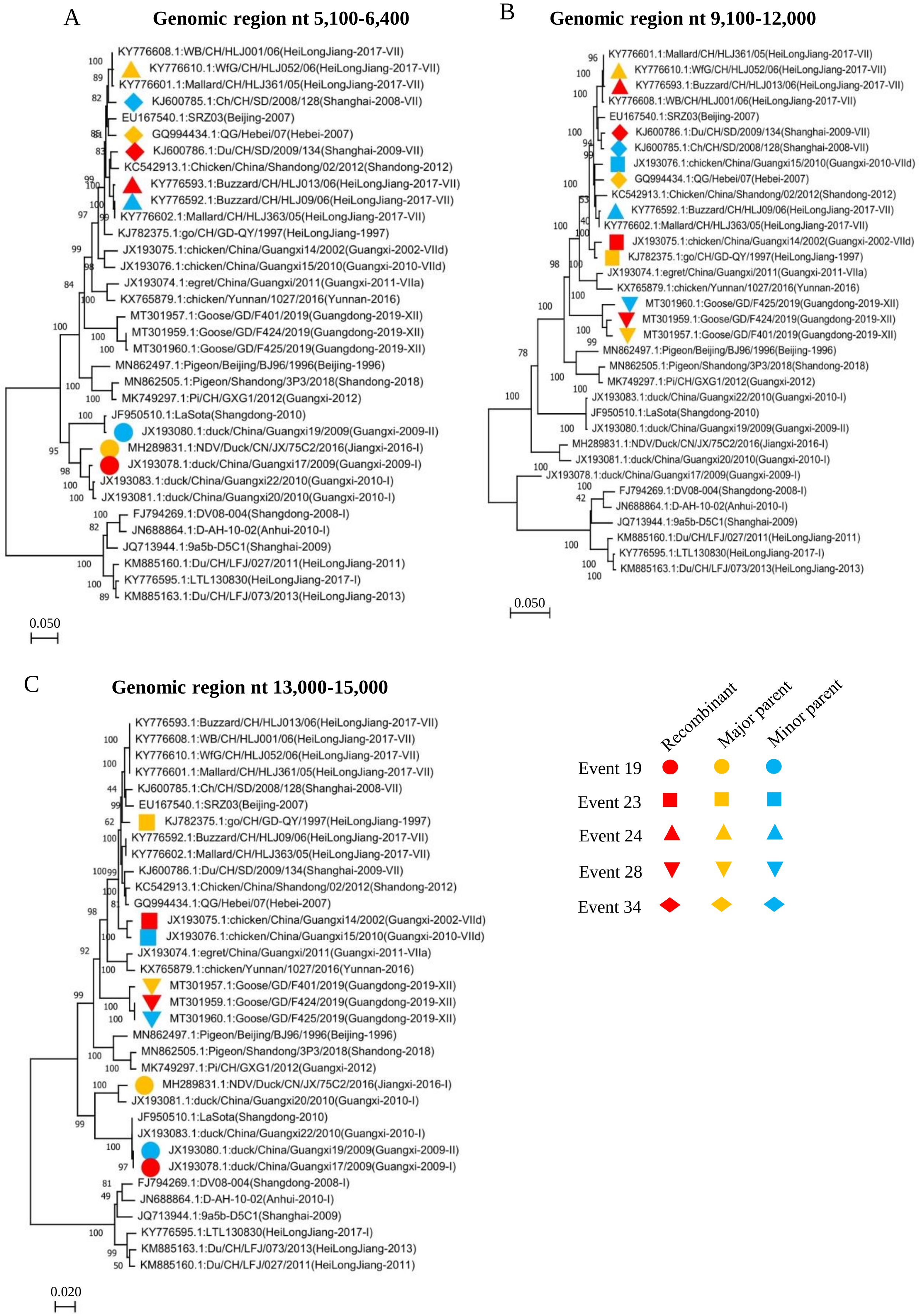

Supplementary Figure S4 SimPlot similarity analysis of NDV full-length genomes involved in recombination events seen in Table 2 and Figure 4 using SimPlot ver.3.5.1, in which Kimura (2-parameter), 200 bp window, and 20 bp Step were used. The recombinant was used as the query in each corresponding event. Major and minor parents are presented with Yellow and Green colors, respectively. The exchanged region in each Event is indicated by a red arrow. Position, nucleotide position of DNV genome relative to corresponding recombinant

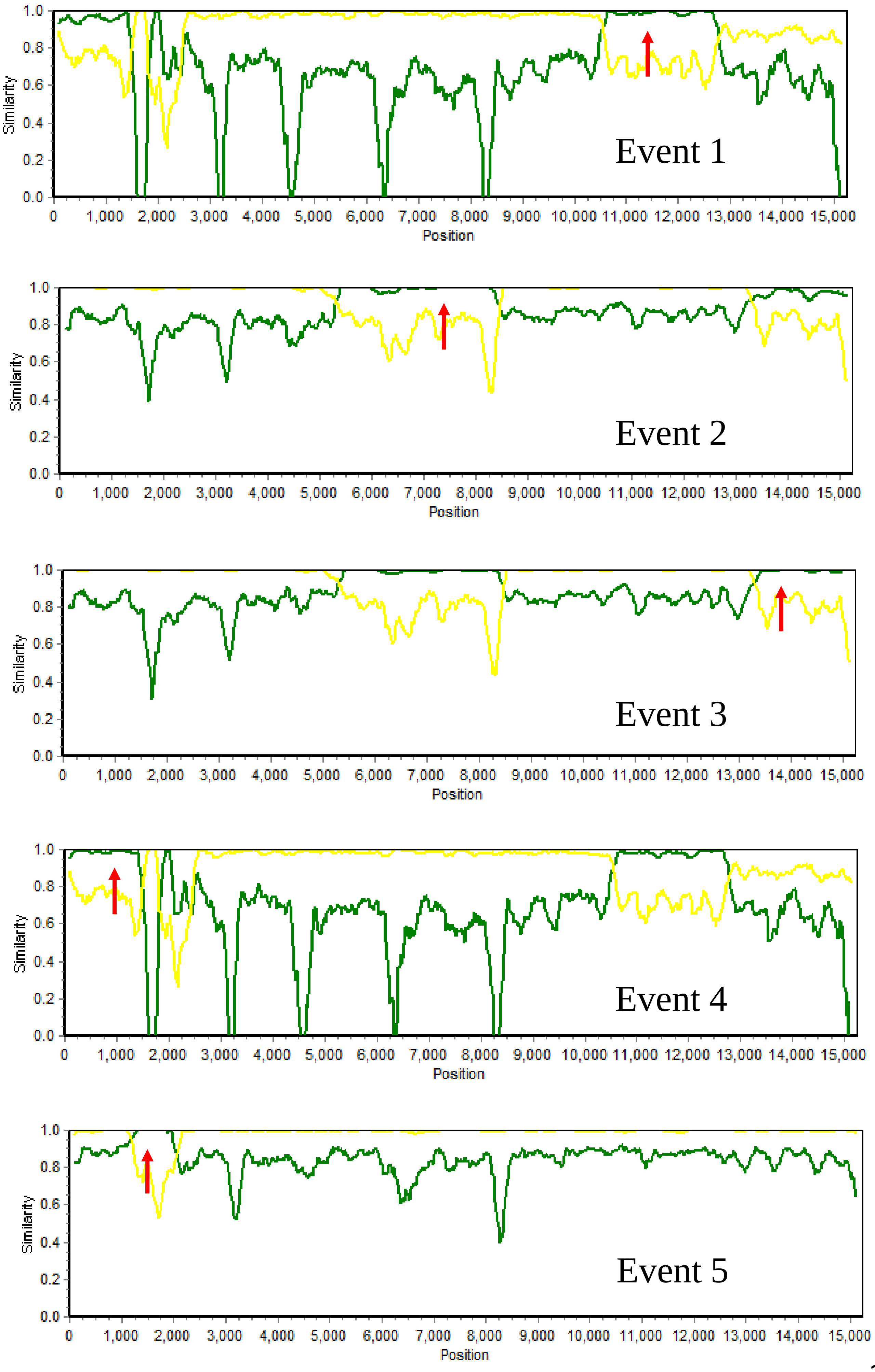

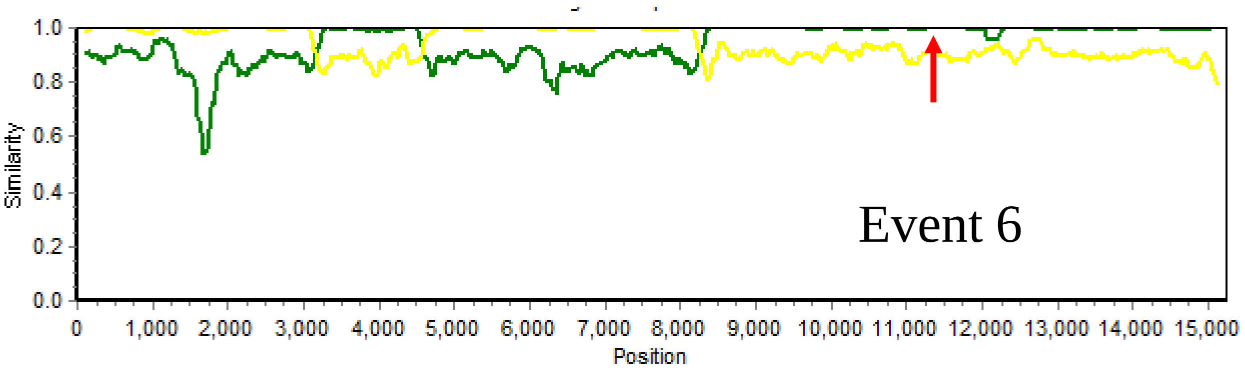
Event 6
Similarity
1.0
0.8
0.6
0.4
0.2
0.0
0
1,000
2,000
3,000
4,000
5,000
6,000
7,000
8,000
9,000
10,000
11,000
12,000
13,000
14,000
15,000
Position

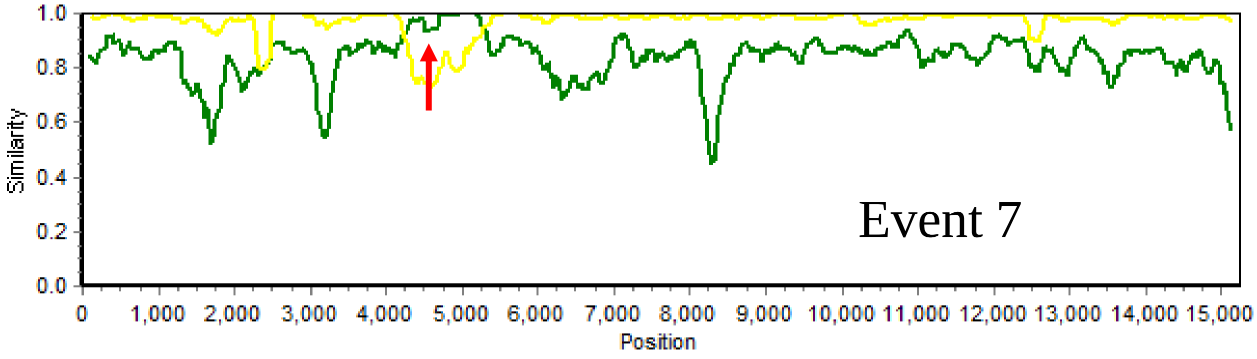
Event 7
Similarity
1.0
0.8
0.6
0.4
0.2
0.0
0
1,000
2,000
3,000
4,000
5,000
6,000
7,000
8,000
9,000
10,000
11,000
12,000
13,000
14,000
15,000
Position

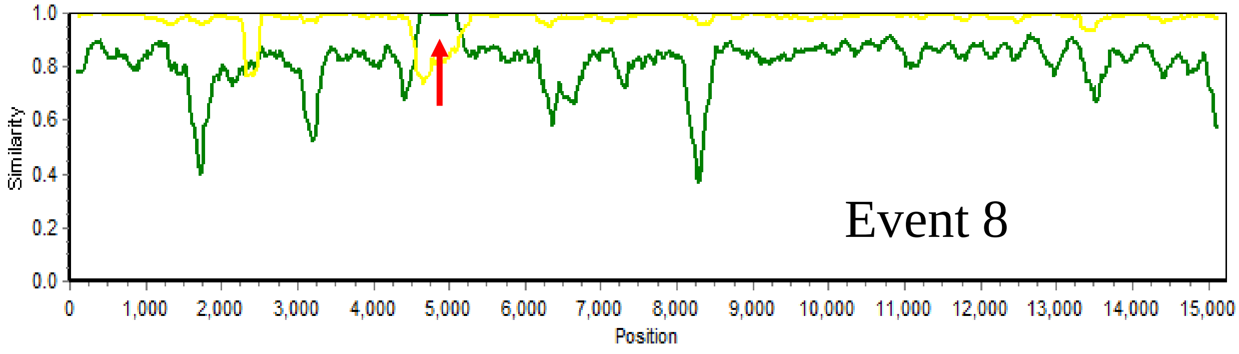
Event 8
Similarity
1.0
0.8
0.6
0.4
0.2
0.0
0
1,000
2,000
3,000
4,000
5,000
6,000
7,000
8,000
9,000
10,000
11,000
12,000
13,000
14,000
15,000
Position

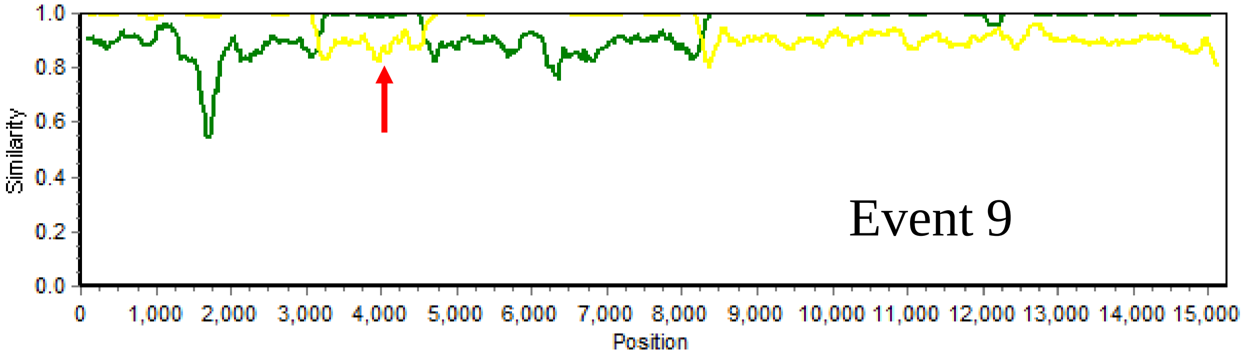
Event 9
Similarity
1.0
0.8
0.6
0.4
0.2
0.0
0
1,000
2,000
3,000
4,000
5,000
6,000
7,000
8,000
9,000
10,000
11,000
12,000
13,000
14,000
15,000
Position

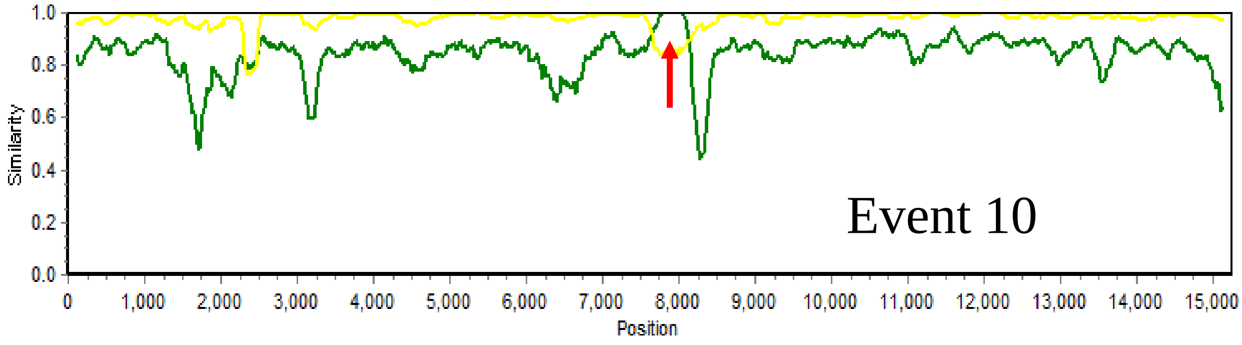
Event 10
Similarity
1.0
0.8
0.6
0.4
0.2
0.0
0
1,000
2,000
3,000
4,000
5,000
6,000
7,000
8,000
9,000
10,000
11,000
12,000
13,000
14,000
15,000
Position

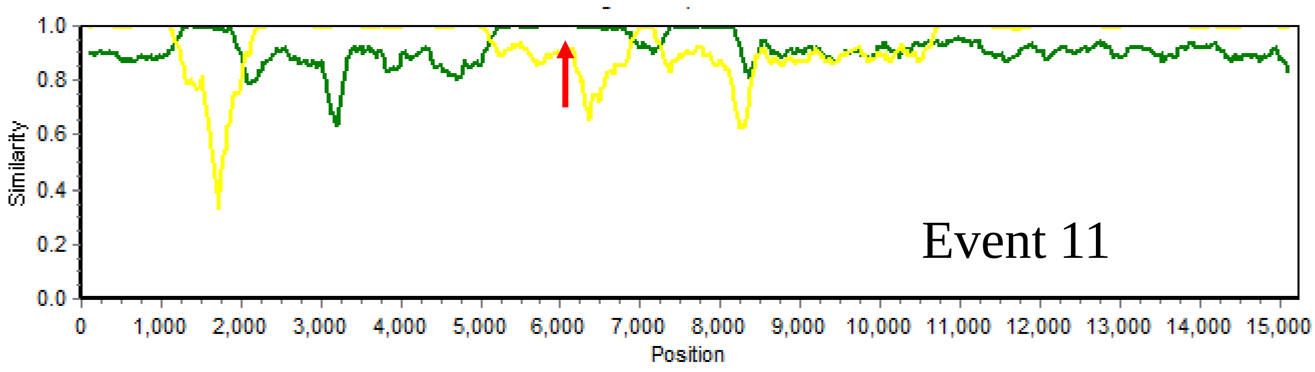
1.0
0.8
0.6
0.4
0.2
0.0
Similarity
Event 11
0
1,000
2,000
3,000
4,000
5,000
6,000
7,000
8,000
9,000
10,000
11,000
12,000
13,000
14,000
15,000
Position

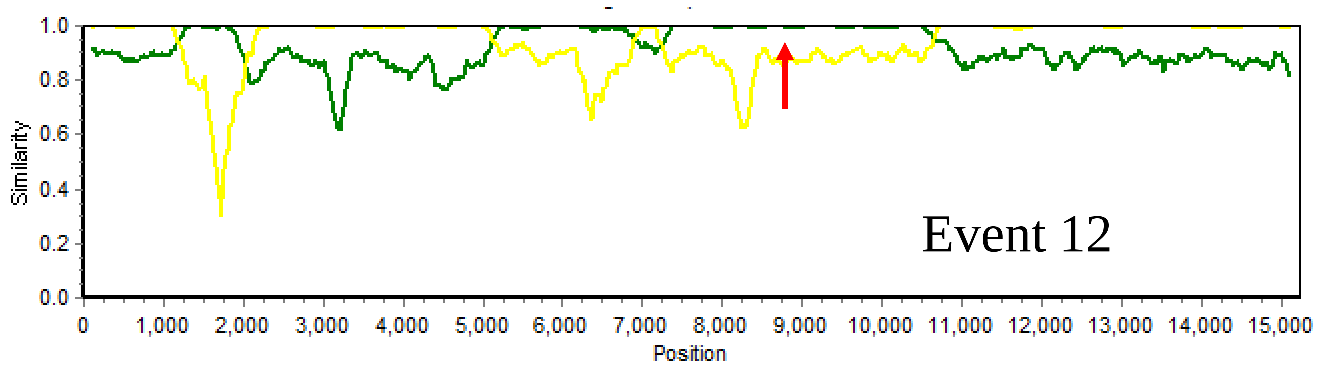
1.0
0.8
0.6
0.4
0.2
0.0
Similarity
Event 12
0
1,000
2,000
3,000
4,000
5,000
6,000
7,000
8,000
9,000
10,000
11,000
12,000
13,000
14,000
15,000
Position

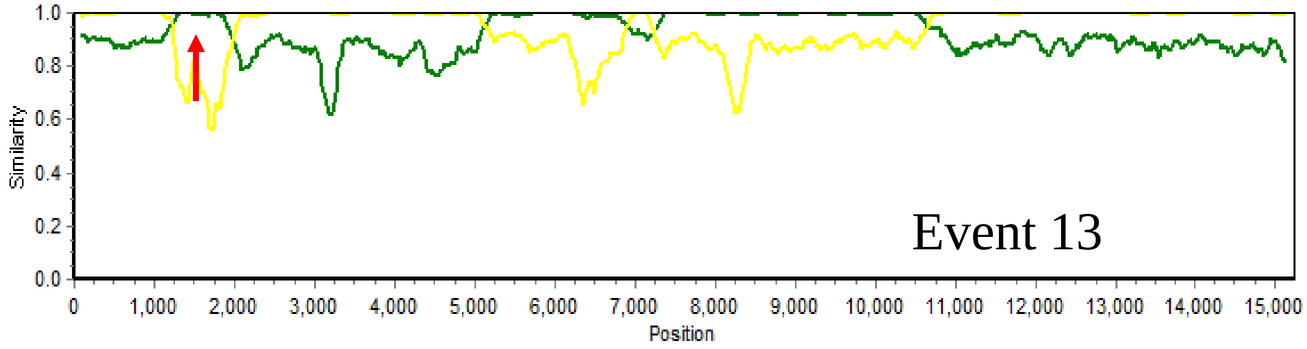
1.0
0.8
0.6
0.4
0.2
0.0
Similarity
Event 13
0
1,000
2,000
3,000
4,000
5,000
6,000
7,000
8,000
9,000
10,000
11,000
12,000
13,000
14,000
15,000
Position

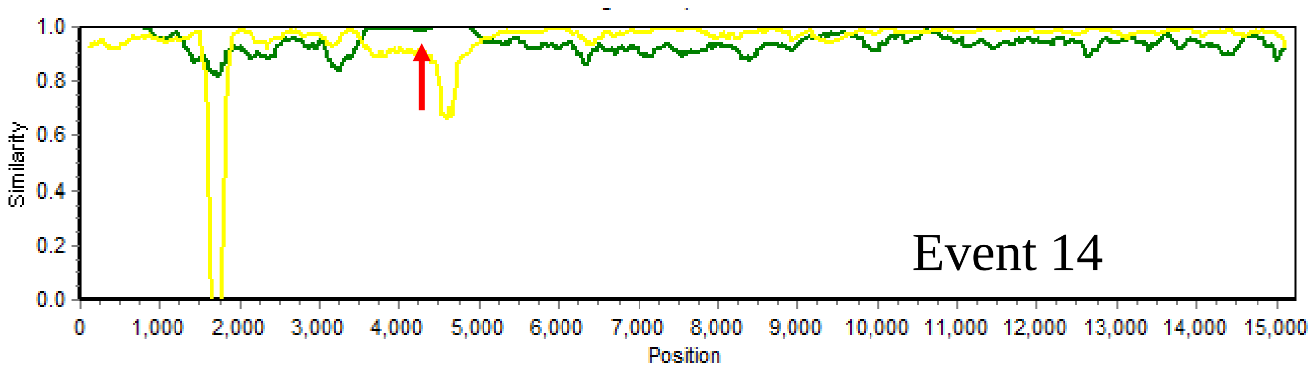
1.0
0.8
0.6
0.4
0.2
0.0
Similarity
Event 14
0
1,000
2,000
3,000
4,000
5,000
6,000
7,000
8,000
9,000
10,000
11,000
12,000
13,000
14,000
15,000
Position

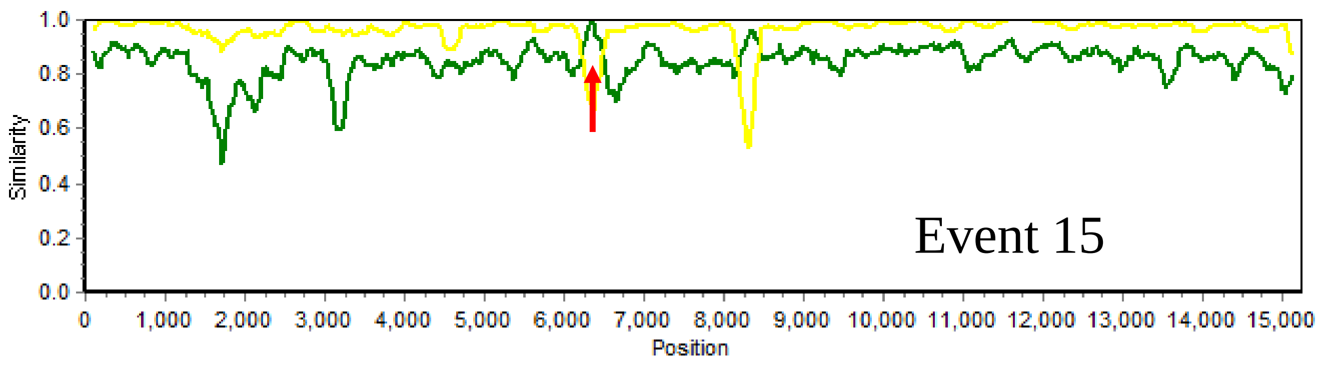
1.0
0.8
0.6
0.4
0.2
0.0
Similarity
Event 15
0
1,000
2,000
3,000
4,000
5,000
6,000
7,000
8,000
9,000
10,000
11,000
12,000
13,000
14,000
15,000
Position

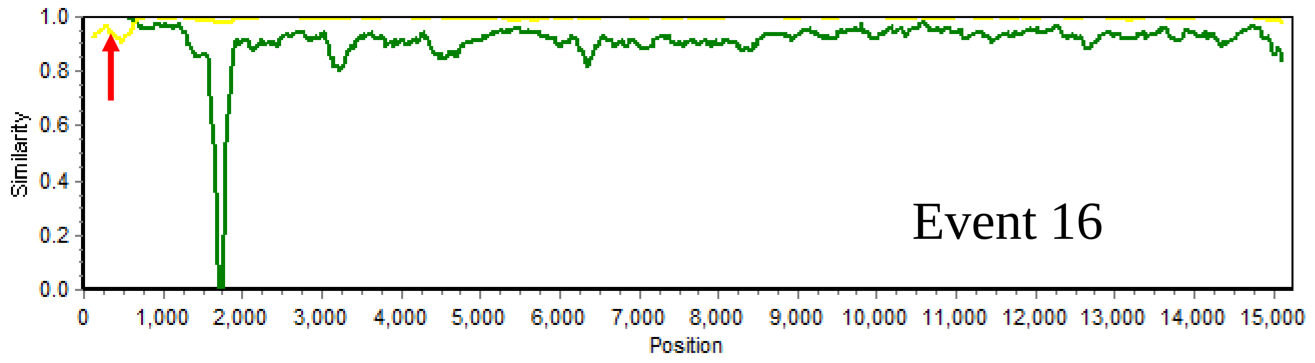
Event 16
Similarity
Position
1.0
0.8
0.6
0.4
0.2
0.0
0 1,000 2,000 3,000 4,000 5,000 6,000 7,000 8,000 9,000 10,000 11,000 12,000 13,000 14,000 15,000

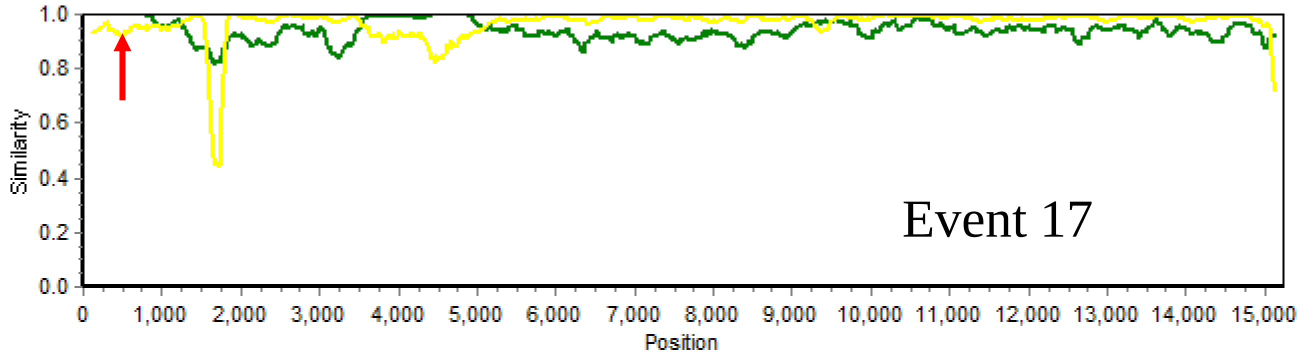
Event 17
Similarity
Position
1.0
0.8
0.6
0.4
0.2
0.0
0 1,000 2,000 3,000 4,000 5,000 6,000 7,000 8,000 9,000 10,000 11,000 12,000 13,000 14,000 15,000

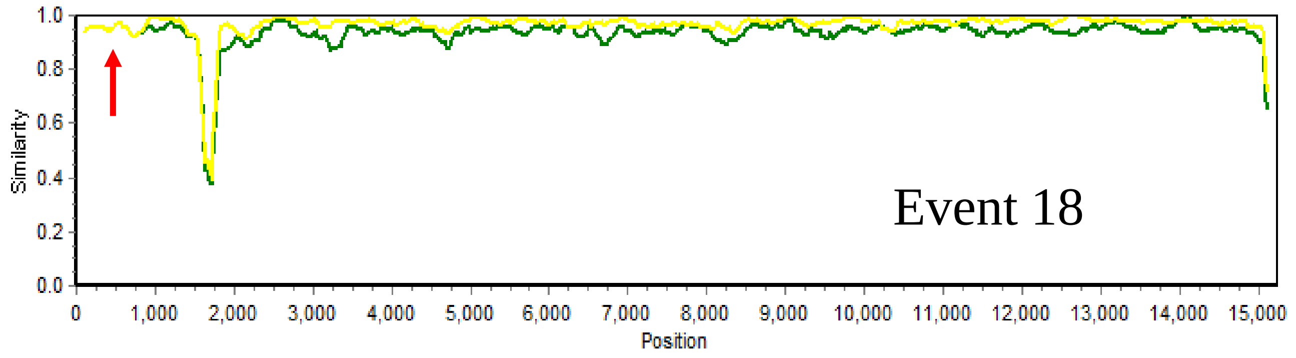
Event 18
Similarity
Position
1.0
0.8
0.6
0.4
0.2
0.0
0 1,000 2,000 3,000 4,000 5,000 6,000 7,000 8,000 9,000 10,000 11,000 12,000 13,000 14,000 15,000

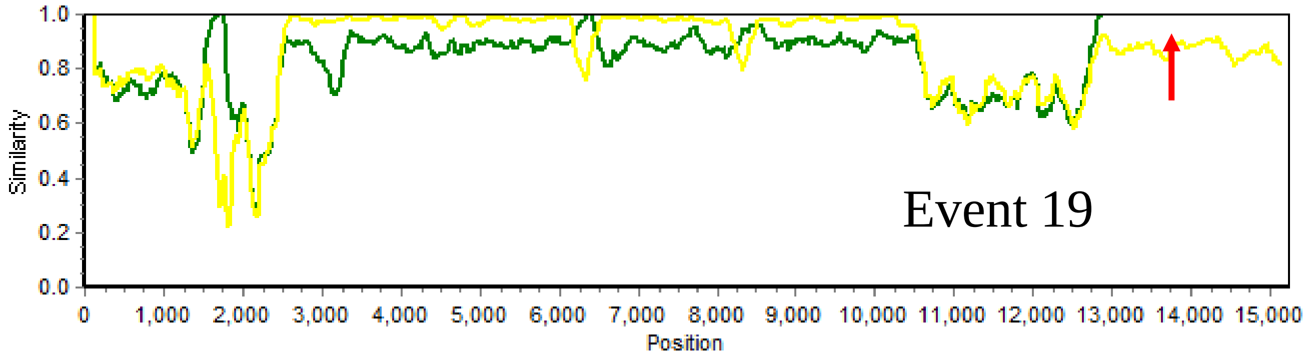
Event 19
Similarity
Position
1.0
0.8
0.6
0.4
0.2
0.0
0 1,000 2,000 3,000 4,000 5,000 6,000 7,000 8,000 9,000 10,000 11,000 12,000 13,000 14,000 15,000

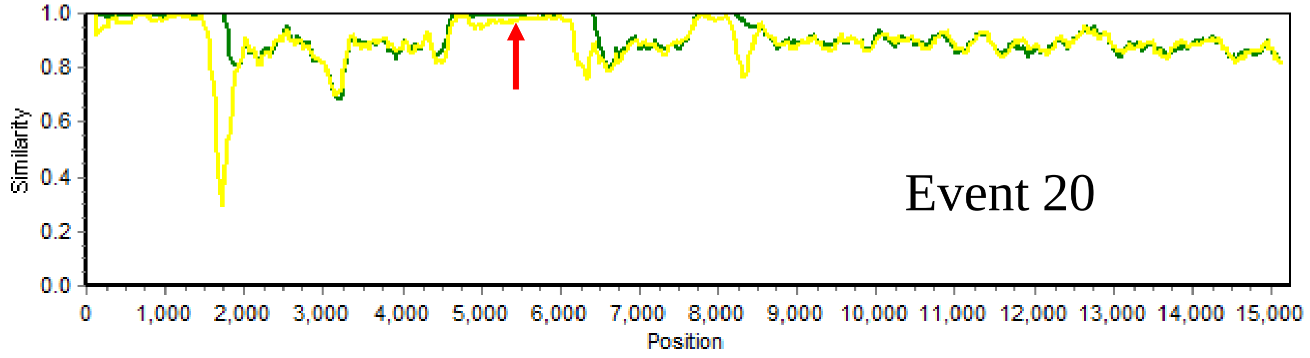
Event 20
Similarity
Position
1.0
0.8
0.6
0.4
0.2
0.0
0 1,000 2,000 3,000 4,000 5,000 6,000 7,000 8,000 9,000 10,000 11,000 12,000 13,000 14,000 15,000

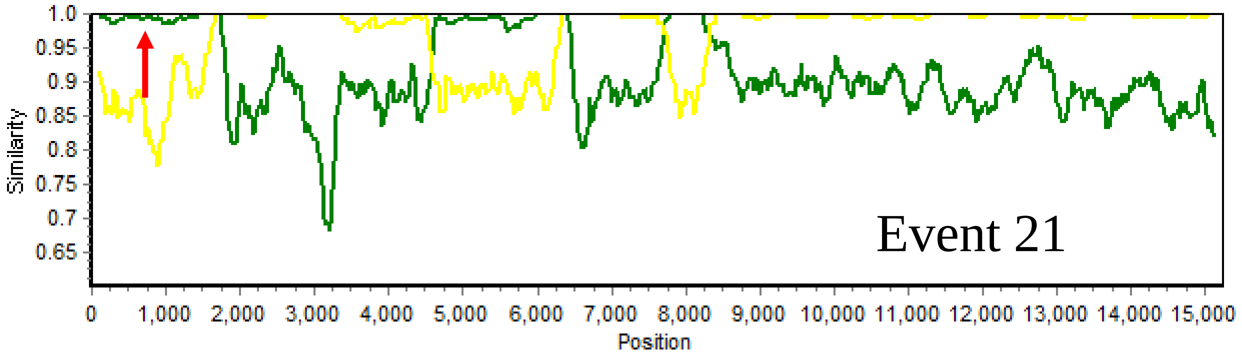
Similarity
1.0
0.95
0.9
0.85
0.8
0.75
0.7
0.65
0 1,000 2,000 3,000 4,000 5,000 6,000 7,000 8,000 9,000 10,000 11,000 12,000 13,000 14,000 15,000
Position
Event 21

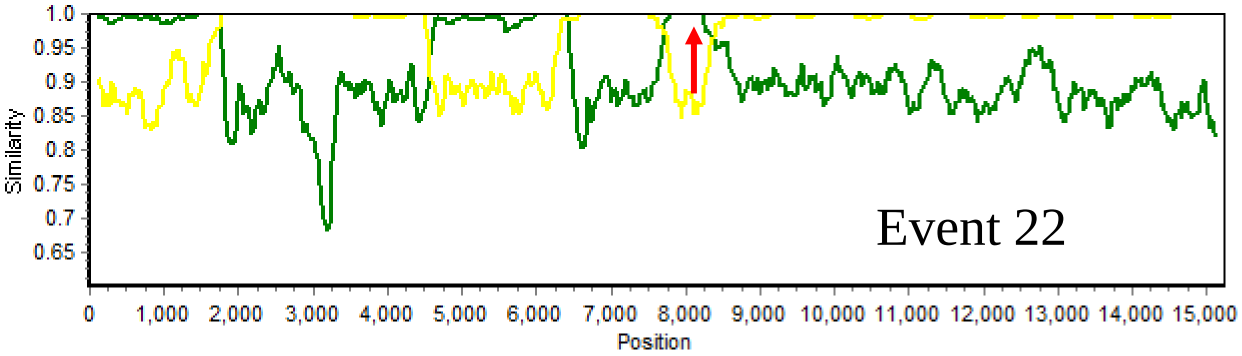
Similarity
1.0
0.95
0.9
0.85
0.8
0.75
0.7
0.65
0 1,000 2,000 3,000 4,000 5,000 6,000 7,000 8,000 9,000 10,000 11,000 12,000 13,000 14,000 15,000
Position
Event 22

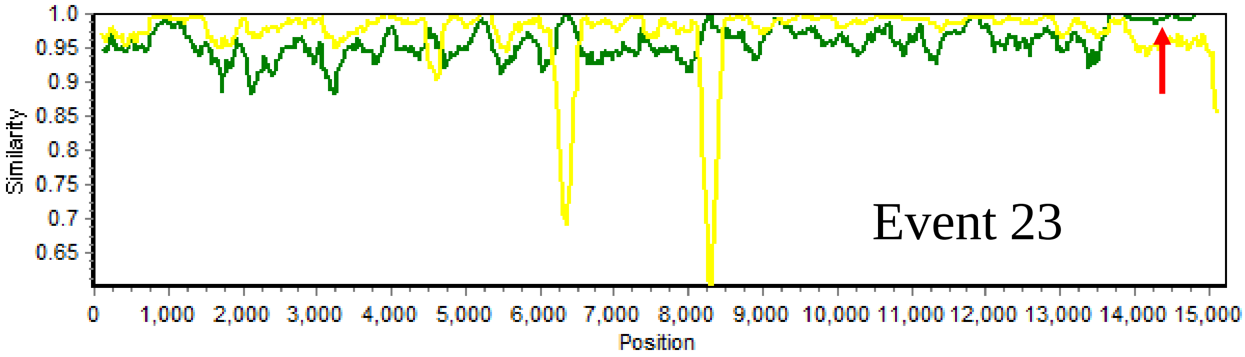
Similarity
1.0
0.95
0.9
0.85
0.8
0.75
0.7
0.65
0 1,000 2,000 3,000 4,000 5,000 6,000 7,000 8,000 9,000 10,000 11,000 12,000 13,000 14,000 15,000
Position
Event 23

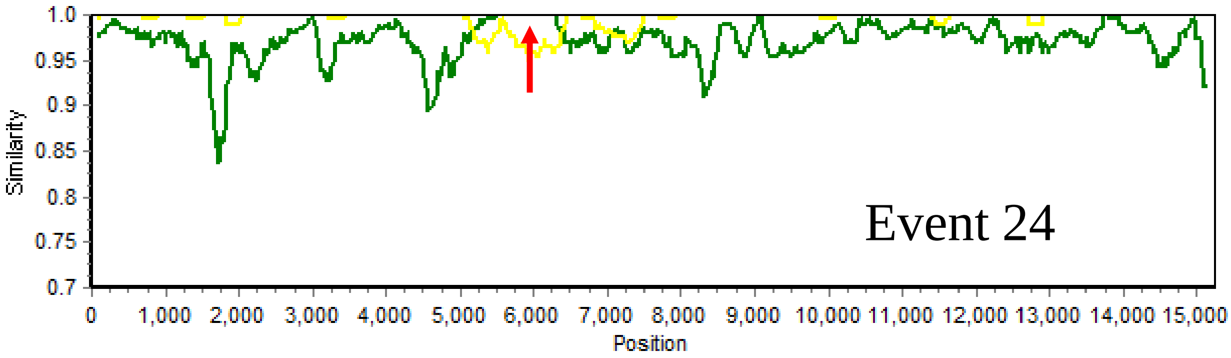
Similarity
1.0
0.95
0.9
0.85
0.8
0.75
0.7
0 1,000 2,000 3,000 4,000 5,000 6,000 7,000 8,000 9,000 10,000 11,000 12,000 13,000 14,000 15,000
Position
Event 24

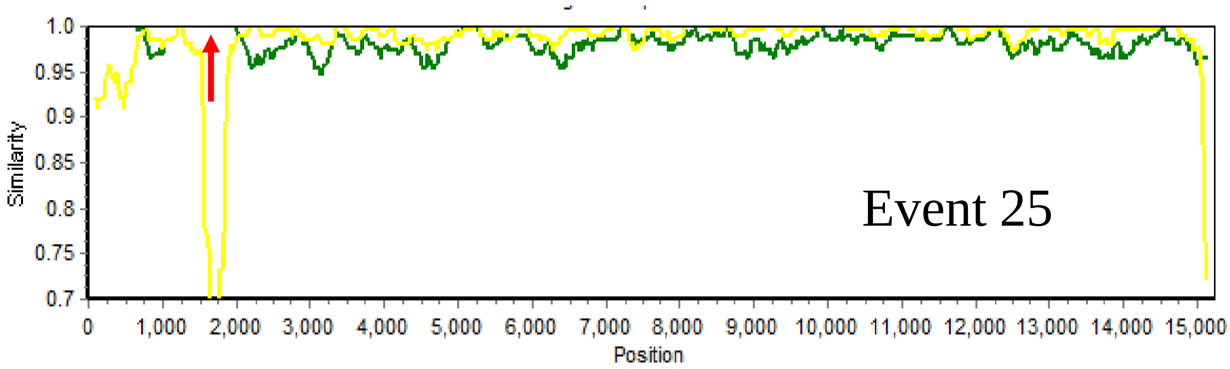
Similarity
1.0
0.95
0.9
0.85
0.8
0.75
0.7
0 1,000 2,000 3,000 4,000 5,000 6,000 7,000 8,000 9,000 10,000 11,000 12,000 13,000 14,000 15,000
Position
Event 25

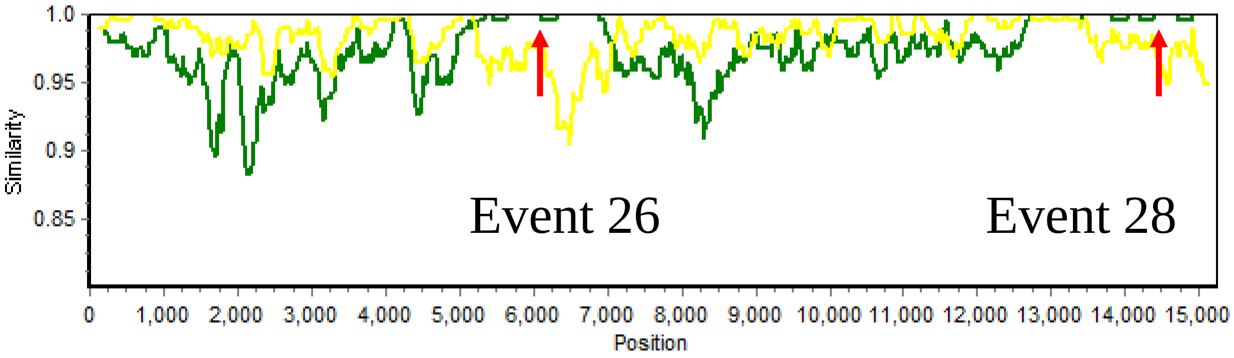
Event 26
Event 28
Similarity
Position

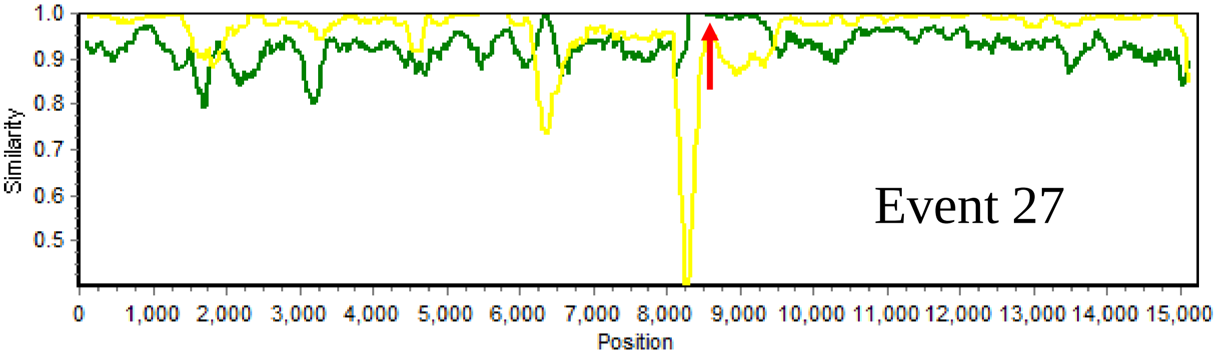
Event 27
Similarity
Position

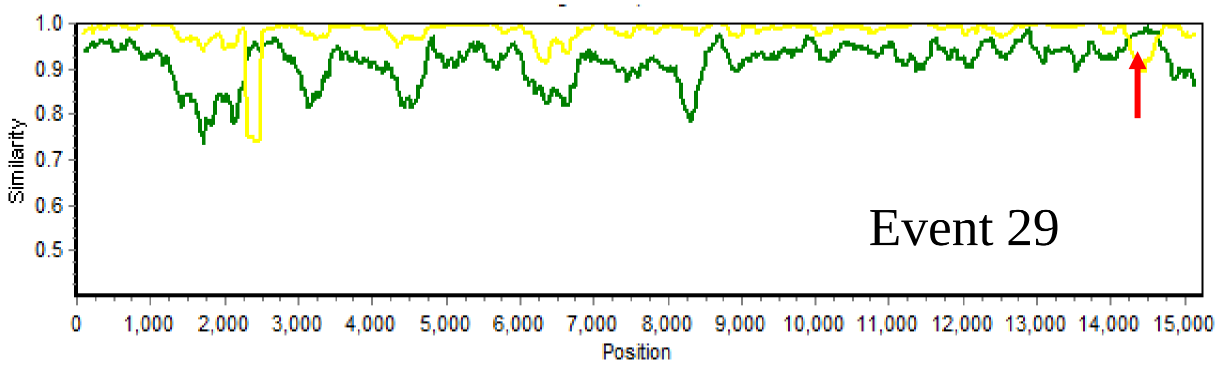
Event 29
Similarity
Position

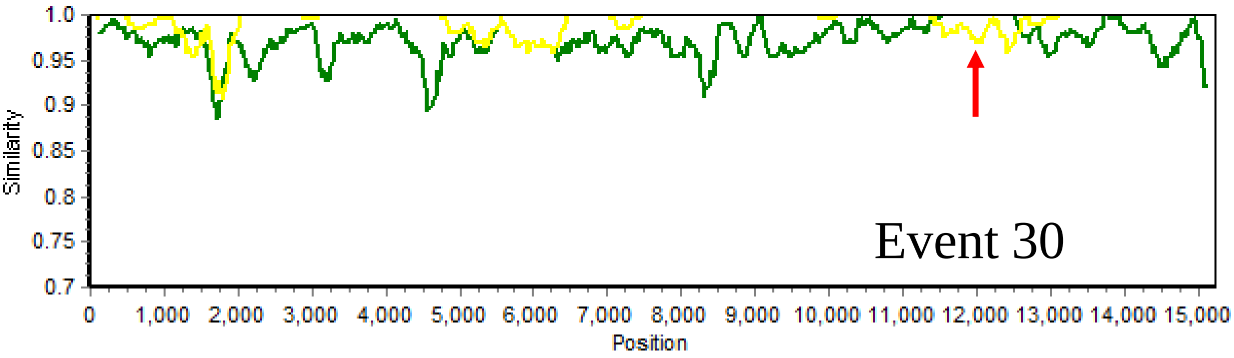
Event 30
Similarity
Position

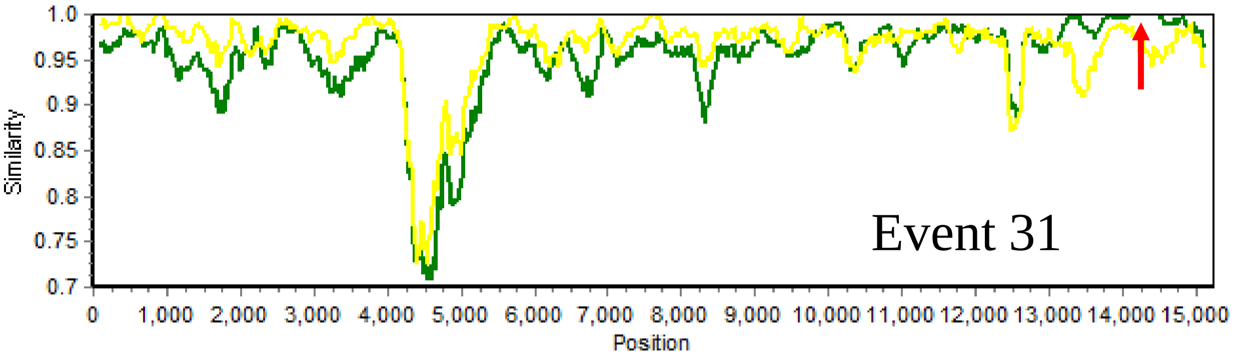
Event 31
Similarity
Position

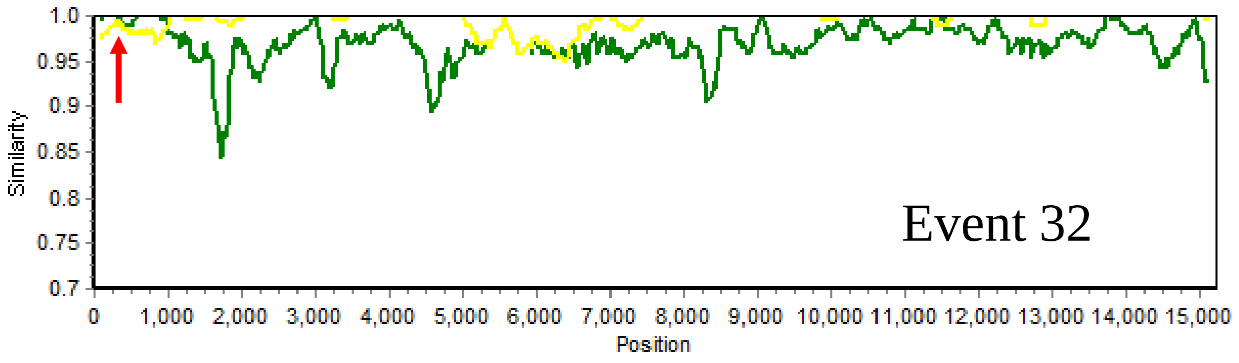
1.0
0.95
0.9
0.85
0.8
0.75
0.7
Similarity
Event 32
0
1,000
2,000
3,000
4,000
5,000
6,000
7,000
8,000
9,000
10,000
11,000
12,000
13,000
14,000
15,000
Position

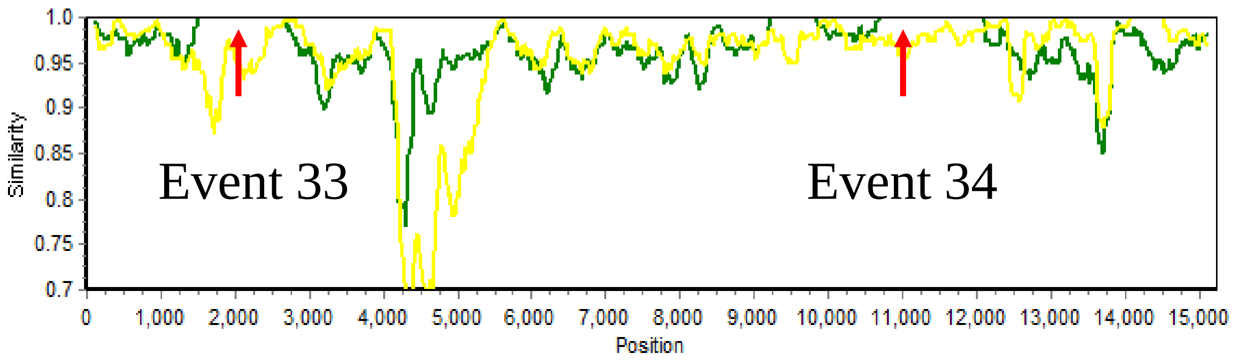
1.0
0.95
0.9
0.85
0.8
0.75
0.7
Similarity
Event 33
Event 34
0
1,000
2,000
3,000
4,000
5,000
6,000
7,000
8,000
9,000
10,000
11,000
12,000
13,000
14,000
15,000
Position
